# First-line immune checkpoint inhibitor plus anti-angiogenic therapy for advanced renal cell carcinoma: a meta-analysis of randomized controlled trials

**DOI:** 10.3389/fphar.2026.1850724

**Published:** 2026-07-27

**Authors:** Zijing Liu, Yu Jiang, Zhihao Zhang, Jie Liu

**Affiliations:** 1 Department of Medical Oncology, Cancer Center, West China Hospital, Sichuan University, Chengdu, China; 2 Department of Breast Surgery, West China Hospital, Sichuan University, Chengdu, China

**Keywords:** anti-angiogenic agents, IMDC risk stratification, immune checkpoint inhibitors, overall survival, renal cell carcinoma

## Abstract

**Background:**

The meta-analysis assesses the efficacy and safety of first-line immune checkpoint inhibitors (ICIs) plus anti-angiogenic therapies versus sunitinib in advanced or metastatic renal cell carcinoma (RCC). The analysis places focus on differential benefits across International Metastatic RCC Database Consortium (IMDC) risk subgroups, ICI classes, and anti-angiogenic drug classes.

**Methods:**

We systematically searched the Cochrane Library, Embase and PubMed for randomized controlled trials (RCTs) comparing ICI plus anti-angiogenic therapy with sunitinib as first-line treatment for advanced RCC. Efficacy measures included progression-free survival (PFS) and overall survival (OS), objective response rate (ORR), disease control rate (DCR). Safety measures assessed grade ≥3 treatment-related adverse events (TRAEs) and TRAEs leading to discontinuation.

**Results:**

Seven RCTs involving 4,977 patients were included. Combination therapy was associated with significant improvements in PFS (0.63, [0.54–0.72]), OS (0.83, [0.77–0.89]), ORR (3.07, [2.01–4.67]), and DCR (2.04, [1.49–2.78]) (all *P* < 0.001). OS benefits were most marked in poor-risk (HR = 0.52) and intermediate-risk (HR = 0.88) patients, while favorable-risk patients showed no significant OS gain (0.97, [0.79–1.18]). Multi-targeted tyrosine kinase inhibitor (TKI)-based regimens provided the greatest PFS and ORR benefits but the highest toxicity, while vascular endothelial growth factor receptor (VEGFR)-selective TKIs showed a balanced efficacy-safety profile. Anti-VEGF monoclonal antibody-based combinations failed to demonstrate significant OS or ORR advantages over sunitinib. Safety analysis showed no significant difference in grade ≥3 TRAEs (1.22, [0.94–1.58]), but did reveal a significantly increased risk of treatment discontinuation (3.34, [2.77–4.03]). Additionally, elevated risks of hepatotoxicity, hypertension, and proteinuria were observed, whereas hematologic toxicities and fatigue were significantly reduced versus sunitinib.

**Conclusion:**

TKI-based ICI combination therapy is superior to sunitinib as first-line treatment for advanced RCC, particularly in intermediate- and poor-risk patients. Anti-angiogenic agent class is the primary determinant of efficacy and tolerability, while ICI class does not influence regimen selection. These findings support a risk-adapted, class-informed approach to optimize therapeutic outcomes in clinical practice.

**Systematic Review registration:**

Identifier CRD420251273931.

## 1 Introduction

According to GLOBOCAN 2020 data, renal cell carcinoma (RCC) is the tenth most prevalent malignancy, accounting for around 430,000 new cases and more than 179,000 deaths each year (Sung et al., 2021). Because early symptoms are typically absent, a substantial proportion of patients present with advanced or metastatic disease at diagnosis. This late presentation leads to a poor clinical outcome, with 5-year overall survival (OS) rates estimated between 0% and 20% (Mekhail et al., 2005). For patients with advanced or unresectable renal cell carcinoma (RCC), systemic therapy remains the mainstay of treatment. Since the approval of sunitinib in 2006, tyrosine kinase inhibitors (TKIs) have been established as the standard first-line treatment for advanced RCC, with sunitinib outperforming interferon-α in terms of median progression-free survival (PFS) (Motzer et al., 2007; Motzer et al., 2006). In recent years, pazopanib, axitinib, and cabozantinib have demonstrated favorable efficacy in individuals with advanced RCC (Motzer et al., 2013; Rini et al., 2011; Choueiri et al., 2016). Everolimus and lenvatinib have also demonstrated promising clinical value in first-line TKI-resistant patients (Motzer et al., 2008; Motzer et al., 2015a). However, for more than a decade after the approval of TKIs, nearly all patients eventually acquired resistance, and none of the systemic therapies evaluated had shown clear superiority over sunitinib until combination therapies incorporating immune checkpoint inhibitors (ICIs) emerged (Motzer et al., 2013).

The advent of ICIs has heralded a transformative era in cancer treatment. RCC demonstrates favorable response potential to immunotherapy. The CheckMate 025 study first demonstrated that nivolumab extended overall survival from 19.6 months with everolimus to 25.0 months in the second-line setting (Motzer et al., 2015b). More importantly, the CheckMate 214 study showed that nivolumab plus ipilimumab extended OS from 26.6 months with sunitinib to 47.0 months in intermediate- and poor-risk patients (Motzer et al., 2022a). The breakthrough advances provided the theoretical foundation for combining ICIs with anti-angiogenic agents. Anti-angiogenic drugs can remodel the tumor microenvironment through vascular normalization and reverse immunosuppressive conditions, thereby boosting the antitumor efficacy of ICIs (Lee et al., 2020).

Recently, multiple randomized controlled trials (RCTs) evaluate the value of ICIs combined with anti-angiogenic agents as first-line management for advanced RCC (Motzer et al., 2026; Rini et al., 2025; Motzer et al., 2024; Choueiri et al., 2025; Motzer et al., 2022b; Yan et al., 2024). However, the results of these trials have revealed several critical inconsistencies and unresolved questions. First, while most trials demonstrated significant PFS benefits, the degree of OS benefit differed considerably across studies. The JAVELIN Renal 101 study extended progression-free survival from 8.5 to 13.9 months, but showed no significant OS difference (Choueiri et al., 2025). However, the CheckMate 9ER study confirmed that improved PFS can lead to significant OS benefits (Motzer et al., 2026). Second, different anti-angiogenic agents appear to yield heterogeneous efficacy outcomes. Multi-targeted TKIs such as cabozantinib and lenvatinib seem to provide greater disease control, whereas bevacizumab-based combinations show more modest effects (Qin et al., 2023). Third, and perhaps most critically, emerging evidence indicates that the advantages of combination therapy may not be uniform across different patient populations stratified by International Metastatic RCC Database Consortium (IMDC) risk criteria (Brown et al., 2020). The CheckMate 214 study notably demonstrated favorable-risk patients did not gain an OS benefit from nivolumab plus ipilimumab compared to sunitinib (Motzer et al., 2022a).

Building on this body of evidence, several important questions remain insufficiently addressed. Prior meta-analyses have largely treated anti-angiogenic agents as a pharmacologically uniform class and have reported IMDC risk subgroup results without formal between-group interaction testing (Tao et al., 2021; Bosma et al., 2022; Yanagisawa et al., 2024). This leaves open two distinct questions: whether efficacy and safety profiles differ meaningfully across mechanistically distinct agent classes, and whether the magnitude of survival benefit is truly modified by IMDC risk category rather than reflecting chance variation in subgroup point estimates. To address these gaps, we conducted an updated and comprehensive meta-analysis of all available RCTs comparing first-line ICI plus anti-angiogenic therapy versus sunitinib in advanced RCC. Critically, and in distinction from prior analyses, this study formally compared three mechanistically distinct classes of anti-angiogenic agents (multi-targeted TKIs, VEGFR-selective TKIs, and anti-VEGF monoclonal antibodies) using pre-specified interaction testing to characterize class-level differences in efficacy and toxicity. In parallel, we performed rigorous interaction analyses across IMDC risk strata to determine whether the magnitude of survival benefit is truly modified by prognostic category, rather than relying on subgroup point estimates alone. Together, these analyses aim to provide a class-informed, risk-adapted evidence base to guide individualized treatment decision-making in clinical practice.

## 2 Materials and methods

This meta-analysis was performed in line with the PRISMA framework and its protocol was registered in the PROSPERO database (CRD420251273931) (Moher et al., 2009).

### 2.1 Search strategy

Literature searches were conducted in PubMed, Embase, and the Cochrane Library. The search strategy consisted of keywords for: (1) RCC; (2) anti-angiogenic agents; and (3) CTLA-4 inhibitors (quavonlimab, tremelimumab, ipilimumab), PD-L1 inhibitors (envafolimab, sugemalimab, durvalumab, avelumab, socazolimab, atezolizumab) and PD-1 inhibitors (penpulimab, nivolumab, toripalimab, sintilimab, tislelizumab, dostarlimab, retifanlimab, serplulimab, camrelizumab, cemiplimab, pembrolizumab). Supplementary Table S1 details the search strategies employed for each database. The search was updated before the final analysis on 1 February 2026.

### 2.2 Inclusion and exclusion criteria

Studies meeting the following criteria were deemed eligible: (1) RCTs, (2) previously untreated patients with metastatic or advanced RCC, (3) patients in the experimental arm received a combination of an anti-angiogenic agent and an ICI, while those in the control arm received sunitinib monotherapy, and (4) studies providing efficacy data on outcomes of interest.

Any study meeting any of the following criteria was excluded from this meta-analysis: (1) non-RCTs; (2) reviews, case reports or meta-analyses; (3) failure to report the predefined outcomes of interest.

Screening of titles and abstracts was performed independently by two reviewers using the established criteria. Full texts were subsequently assessed, and any disputes were settled by consulting a third reviewer. This same approach was also used for quality assessment and data collection.

### 2.3 Risk of bias assessment, outcome definition, and data collection

Methodological quality of the included RCTs was evaluated using the Cochrane risk of bias tool (Higgins et al., 2011). Bias evaluation for each trial encompassed seven domains: random sequence generation, allocation concealment, blinding of participants and personnel, blinding of outcome assessment, completeness of outcome data, selective reporting, and other biases (Higgins et al., 2011). Based on the risk of bias assessment, the included studies were judged to be of low, high, or moderate quality.

Information was collected from each trial using a standardized data collection tool. The extracted information covered the outcomes of interest, encompassing OS, PFS, ORR, DCR, grade ≥3 TRAEs, and TRAEs leading to treatment discontinuation. Extracted results comprised event counts, HR, 95% CIs, and *P*-values. Post-progression treatment patterns, including subsequent ICI and TKI use rates in each arm, were also extracted to contextualize OS interpretation.

### 2.4 Statistical analysis

Stata/MP 17.0 (Revision 14 June 2021; College Station, TX, United States) was used to conduct statistical analyses. Publication bias was assessed using funnel plots and Egger’s test. If significant bias was detected for any outcome measure, a leave-one-out sensitivity analysis was conducted to identify studies with substantial influence, and the non-parametric “trim-and-fill” method was employed to estimate the potential impact of missing studies on the pooled effect size. Heterogeneity across trials was quantitatively assessed using the I^2^ statistic; values exceeding 50% were considered to indicate substantial heterogeneity. A random-effects model was utilized in the presence of substantial heterogeneity, whereas a fixed-effects model was applied when heterogeneity was low. Binary endpoints were reported as OR with 95% CIs, including ORR, DCR, and the incidence of grade ≥3 TRAEs and TRAEs leading to treatment discontinuation. Survival outcomes are presented as HRs (and 95% CIs) for PFS and OS. Subgroup analyses were prespecified to evaluate potential factors that may modify the treatment effect and to test the stability of the primary results. To evaluate the impact of each study on the pooled results, sensitivity analyses were carried out by iteratively omitting one study at a time. Statistical significance for all tests was set at a two-tailed (*P* < 0.05).

## 3 Results

### 3.1 Study selection

**FIGURE 1:**
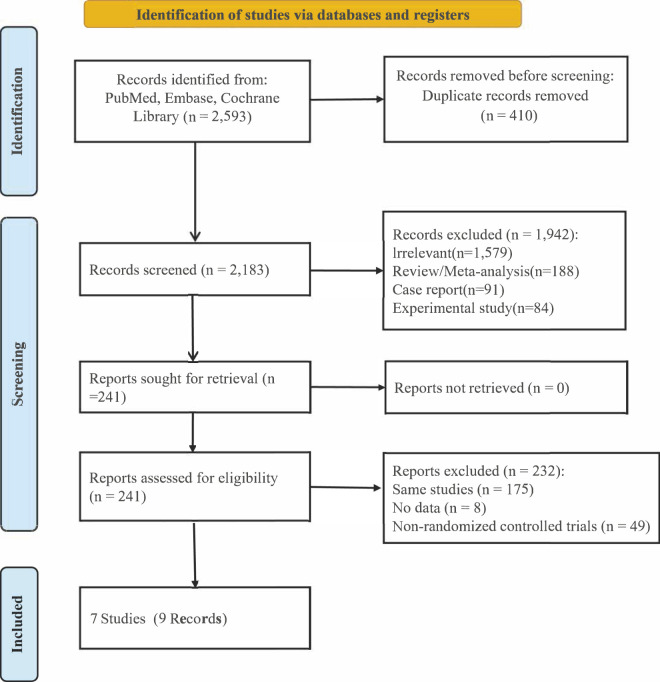
Preferred reporting items for systematic reviews and meta-analyses (PRISMA) flowchart of retrieved studies.

Figure 1 outlines the screening and selection of studies. A total of 2,593 records were identified through database searches in PubMed, Embase, and the Cochrane Library. After excluding 410 duplicates, 2,183 articles underwent title and abstract screening. Of these, 1,942 were excluded for various reasons: 1,579 were unrelated to the research topic, 188 were reviews or meta-analyses, 91 were case reports, and 84 were experimental studies. The remaining 241 full-text articles underwent eligibility assessment, of which 232 were excluded due to duplication or overlap (175), not being randomized controlled trials (49), or lacking relevant outcome data (8). Two articles originally considered duplicates were retained for data extraction, as they represented prior reports of the IMmotion151 and KEYNOTE-426 trials, which provided necessary data for subsequent analyses (Rini et al., 2019; Powles et al., 2020). Ultimately, seven studies (nine records) were included (Motzer et al., 2026; Rini et al., 2025; Motzer et al., 2024; Choueiri et al., 2025; Motzer et al., 2022b; Yan et al., 2024; Rini et al., 2019; Powles et al., 2020; Zhou et al., 2025). Table 1 lists the main features of the studies included in this analysis, and quality assessment outcomes can be found in Supplementary Table S2.

**TABLE 1:** Characteristics of included studies.

Study	Year	Recruitment period	Country	Sample size	Intervention arm (n)	Control arm (n)	Median PFS (months)	PFS HR (95% CI)	Median OS (months)	OS HR (95% CI)
CheckMate 9ER	2025	11 September 2017 – 14 May 2019	Multinational (18 countries)	651	Nivolumab + cabozantinib (323)	Sunitinib (328)	16.4 vs. 8.3	0.58 (0.49, 0.70)	46.5 vs. 35.5	0.79 (0.65, 0.96)
CLEAR	2024	13 October 2016 – 24 July 2019	Multinational (20 countries)	712	Lenvatinib + pembrolizumab (355)	Sunitinib (357)	23.9 vs. 9.2	0.47 (0.38, 0.57)	53.7 vs. 54.3	0.79 (0.63, 0.99)
ETER100	2025	25 August 2020 – 6 February 2023	China	531	Benmelstobart + anlotinib (266)	Sunitinib (265)	19.0 vs. 9.8	0.53 (0.42, 0.67)	NR	0.66 (0.48, 0.92)
IMmotion151	2022	20 May 2015 - 12 Oct 2016	Multinational (21 countries)	915	Atezolizumab + bevacizumab (454)	Sunitinib (461)	11.2 vs. 8.4	0.83 (0.70, 0.97)	36.1 vs. 35.3	0.91 (0.76, 1.08)
JAVELIN renal 101	2024	29 March 2016–19 December 2017	Multinational (29 countries)	886	Avelumab + axitinib (442)	Sunitinib (444)	13.9 vs. 8.5	0.66 (0.57, 0.77)	44.8 vs. 38.9	0.88 (0.75, 1.04)
KEYNOTE-426	2025	24 October 2016 – 24 January 2018	Multinational (16 countries)	861	Pembrolizumab + axitinib (432)	Sunitinib (429)	15.4 vs. 11.1	0.69 (0.59, 0.81)	47.2 vs. 40.8	0.84 (0.71, 0.99)
RENOTORCH	2023	23 August 2020 – 23 September 2022	China	421	Toripalimab + axitinib (210)	Sunitinib (211)	18.0 vs. 9.8	0.65 (0.49, 0.86)	NR vs. 26.8	0.61 (0.40, 0.92)

Abbreviation: PFS, progression-free survival; OS, overall survival; NR, not reached.

### 3.2 Progression-free survival and overall survival

**FIGURE 2:**
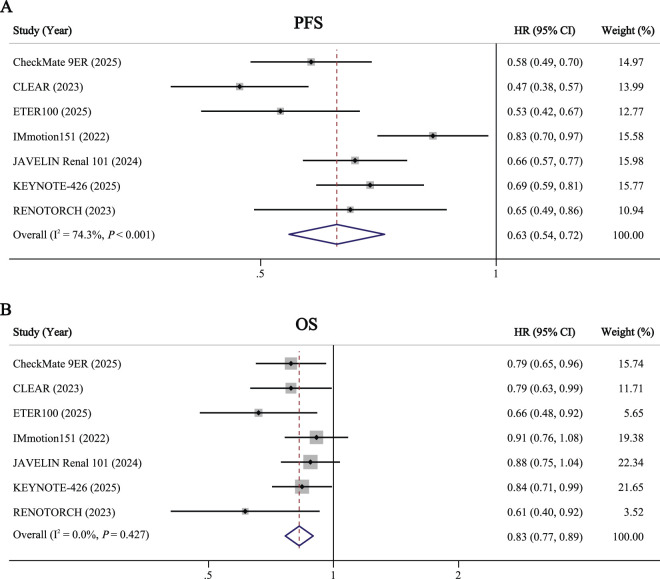
Forest plots of **(A)** PFS and **(B)** OS for ICIs plus anti-angiogenic therapy versus sunitinib in first-line treatment of advanced RCC. Abbreviations: PFS, progression-free survival; OS, overall survival; ICIs, immune checkpoint inhibitors; HR, hazard ratio; CI, confidence interval; RCC, renal cell carcinoma.

This meta-analysis demonstrated significant survival benefits for first-line ICIs combined with anti-angiogenic agents relative to sunitinib in the advanced RCC population. The combination therapy was associated with a 37% lower hazard of disease progression (0.63 [95% CI: 0.54–0.72]; *P* < 0.001). Additionally, the risk of mortality was reduced by 17% (0.83 [95% CI: 0.77–0.89]; *P* < 0.001) (Figure 2).

**FIGURE 3:**
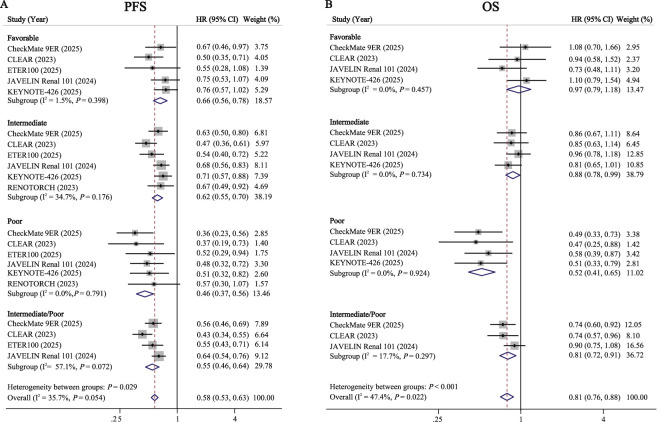
Forest plots of subgroup analyses for **(A)** PFS and **(B)** OS based on IMDC risk levels in advanced RCC. Abbreviations: PFS, progression-free survival; OS, overall survival; ICIs, immune checkpoint inhibitors; IMDC, International Metastatic RCC Database Consortium; RCC, renal cell carcinoma; HR, hazard ratio; CI, confidence interval.

Subgroup analyses by IMDC risk category revealed differential magnitudes of benefit in Figure 3. For PFS, the combination therapy substantially lowered the hazard of progression across all subgroups, with the most pronounced reduction observed in poor-risk patients (0.46 [95% CI: 0.37–0.56]), followed by intermediate (0.62 [95% CI: 0.55–0.70]) and favorable patients (0.66 [95% CI: 0.56–0.78]). The difference in treatment effect between poor and favorable patients was statistically significant (*P* for interaction = 0.006), as was the difference between poor and intermediate patients (*P* for interaction = 0.015), while no significant difference was observed between favorable and intermediate patients (*P* for interaction = 0.515) or between favorable and combined intermediate or poor patients (*P* for interaction = 0.105). Notably, IMDC-based stratification substantially reduced within-subgroup PFS heterogeneity relative to the overall analysis, with within-subgroup I^2^ values of 1.5% in favorable-risk, 34.7% in intermediate-risk, and 0.0% in both poor-risk and intermediate/poor combined subgroups. For OS, no significant survival benefit was observed in favorable-risk patients (0.97 [95% CI: 0.79–1.18]). In contrast, significant OS benefits were observed in both intermediate-risk (0.88 [95% CI: 0.78–0.99]) and poor-risk patients (0.52 [95% CI: 0.41–0.65]), with a statistically significant difference in treatment effect between these two higher-risk subgroups (*P* for interaction <0.001).

**FIGURE 4:**
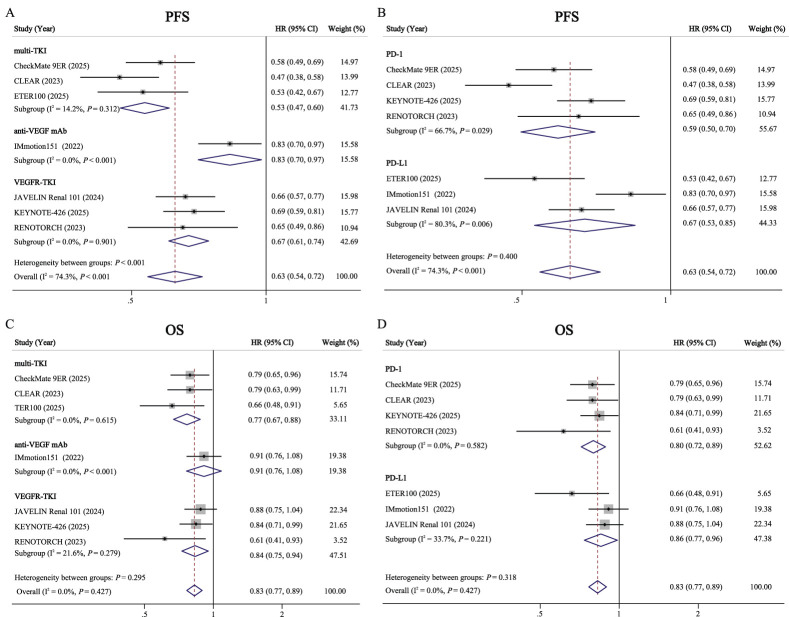
Forest plots of subgroup analyses for **(A,C)** anti-angiogenic drug types and **(B,D)** ICIs types on PFS and OS in advanced RCC. Abbreviations: PFS, progression-free survival; OS, overall survival; ICIs, immune checkpoint inhibitors; RCC, renal cell carcinoma; HR, hazard ratio; CI, confidence interval.

Subgroup analyses of PFS and OS stratified by anti-angiogenic drug class and ICI type are presented in Figure 4. Stratification by the type of anti-angiogenic drug demonstrated distinct efficacy profiles, particularly for PFS. For PFS, the treatment effect varied significantly across different agent classes (*P* for interaction <0.050). The greatest risk reduction was observed with regimens combining ICIs with multi-targeted TKIs (0.53 [95% CI: 0.47–0.60]; I^2^ = 14.2%). This was followed by combinations with VEGFR-selective TKIs (0.67 [95% CI: 0.61–0.74]; I^2^ = 0.0%), which in turn showed a more pronounced benefit than those with anti-VEGF mAbs (0.83 [95% CI: 0.71–0.98]). Importantly, class-based stratification substantially reduced within-subgroup PFS heterogeneity relative to the overall analysis: within-subgroup I^2^ was 14.2% for multi-TKI combinations, 0.0% for both VEGFR-TKI combinations and the anti-VEGF mAb subgroup. In contrast, for OS, the benefit was more uniform across these classes, with no statistically significant difference observed between multi-TKI-based (0.77 [95% CI: 0.67–0.88]) and VEGFR-TKI-based regimens (0.84 [95% CI: 0.75–0.94]) (*P* for interaction = 0.310). The single anti-VEGF mAb-based regimen failed to demonstrate a significant OS advantage (0.91 [95% CI: 0.76–1.08]). Furthermore, subgroup analysis by the type of ICI indicated that both classes of ICI-based regimens conferred significant and comparable PFS benefits (*P* for interaction = 0.400). A similar pattern was observed for OS, with no meaningful distinction between the two ICI classes (*P* for interaction = 0.318).

Additional subgroup analyses from Supplementary Table S3 further supported the robustness of the PFS benefit across demographics (age, sex), baseline clinical features (Eastern Cooperative Oncology Group performance status, sarcomatoid features, prior nephrectomy, metastatic sites). Subgroup analyses by PD-L1 expression status, assessed by both CPS and IC scoring methodologies, consistently showed no statistically significant interaction with treatment effect for either PFS or OS (all *P* for interaction >0.050).

Post-progression treatment data were extracted from all seven trials and are summarized in Supplementary Table S6. A consistent and marked asymmetry was observed in subsequent ICI use between arms: in the sunitinib control arms, the proportion of patients receiving subsequent ICI-based therapy ranged from 23.2% (ETER100) to 81.0% (CheckMate 9ER), whereas rates in the corresponding combination arms were substantially lower (9.5%–35.7%).

### 3.3 Objective response rate and disease control rate

Supplementary Figure S1 displays the forest plots for ORR and DCR. The pooled analysis showed that ORR improved substantially (3.07 [95% CI: 2.01–4.67]; *P* < 0.001), with highly inconsistent results across studies (I^2^ = 92.1%). Similarly, DCR was robustly improved (2.04 [95% CI: 1.49–2.78]; *P* < 0.001), although marked inconsistency persisted (I^2^ = 76.4%).

To explore the origins of heterogeneity, subgroup analyses were conducted according to IMDC risk criteria (Supplementary Figure S2). We performed pairwise comparisons across the IMDC risk subgroups. The ORR benefit associated with combination therapy was comparable between groups. Specifically, the benefit in the favorable risk subgroup (3.34 [95% CI: 2.20–5.06]) did not differ significantly from that in the intermediate (3.91 [95% CI: 2.50–6.12]; *P* for interaction = 0.610), poor (5.52 [95% CI: 3.06–9.96]; *P* for interaction = 0.172), or intermediate/poor combined subgroups (4.60 [95% CI: 2.61–8.08]; *P* for interaction = 0.371). IMDC-based stratification did not resolve ORR heterogeneity: substantial within-subgroup I^2^ persisted in the intermediate-risk subgroup (I^2^ = 78.1%), with moderate residual heterogeneity also observed in the poor-risk subgroup (I^2^ = 31.1%).

Subgroup analysis by drug class provided further clarification and revealed a distinct efficacy gradient (Supplementary Figure S3). For ORR, the benefit was most marked with ICI plus multi-targeted TKI combinations (4.70 [95% CI: 3.01–7.33]), followed by ICI plus VEGFR-TKI regimens (2.76 [95% CI: 2.28–3.34]), while the benefit from ICI plus anti-VEGF mAbs was not significant (1.16 [95% CI: 0.88–1.52]). Pairwise comparisons confirmed that the ORR benefit of multi-TKI-based combinations was significantly greater than that of both VEGFR-TKI-based (*P* for interaction = 0.031) and anti-VEGF mAb-based regimens (*P* for interaction <0.001). The benefit with VEGFR-TKI combinations was also significantly superior to that with anti-VEGF mAb (*P* for interaction <0.001). At the between-class level, IMmotion151 diverged markedly from all regimens. At the within-class level, heterogeneity was substantially reduced in the VEGFR-TKI subgroup (I^2^ = 14.5%) and was absent by definition in the single-trial anti-VEGF mAb subgroup (I^2^ = 0.0%). However, substantial residual heterogeneity persisted within the multi-TKI subgroup (I^2^ = 80.3%), driven by markedly differing response magnitudes across cabozantinib (OR = 3.33), lenvatinib (OR = 4.28), and anlotinib-based regimens (OR = 7.52). For DCR, a significant benefit was observed across all drug classes, though the magnitude differed. Multi-TKI-based combinations again showed the highest point estimate (3.23 [95% CI: 2.47–4.21]), which was significantly greater than that of anti-VEGF mAb-based therapy (1.23 [95% CI: 0.91–1.65]; *P* for interaction <0.001) and VEGFR-TKI-based therapy (1.66 [95% CI: 1.33–2.06]; *P* for interaction <0.001). The difference in DCR benefit between multi-TKI and VEGFR-TKI combinations was statistically significant, as was the difference between multi-TKI and anti-VEGF mAb combinations (both *P* for interaction <0.001). Class-based stratification completely resolved within-subgroup heterogeneity for DCR, with I^2^ reaching 0.0% across all three drug classes.

In contrast, subgroup analysis by ICI type showed no statistically significant difference in the magnitude of benefit for either ORR (*P* for interaction = 0.933) or DCR (*P* for interaction = 0.444).

### 3.4 Grade ≥3 TRAEs and TRAEs leading to discontinuation

**FIGURE 5:**
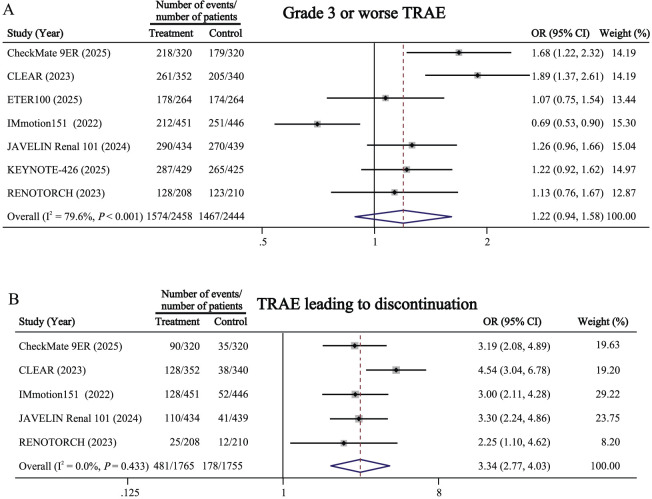
Forest plots of TRAEs for ICIs plus anti-angiogenic therapy versus sunitinib in advanced RCC: **(A)** Grade 3 or worse TRAEs and **(B)** TRAEs leading to treatment discontinuation. Abbreviations: TRAEs, treatment-related adverse events; ICIs, immune checkpoint inhibitors; OR, odds ratio; CI, confidence interval; RCC, renal cell carcinoma.

As shown in Figure 5, the overall odds of grade ≥3 TRAEs were numerically higher, though not statistically significant (1.22 [95% CI: 0.94–1.58]). Heterogeneity was substantial (I^2^ = 79.6%, *P* < 0.001). In contrast, TRAEs leading to treatment discontinuation occurred more than twice as often in the combination arms (3.34 [95% CI: 2.77–4.03]; I^2^ = 0.0%, *P* < 0.001).

**FIGURE 6:**
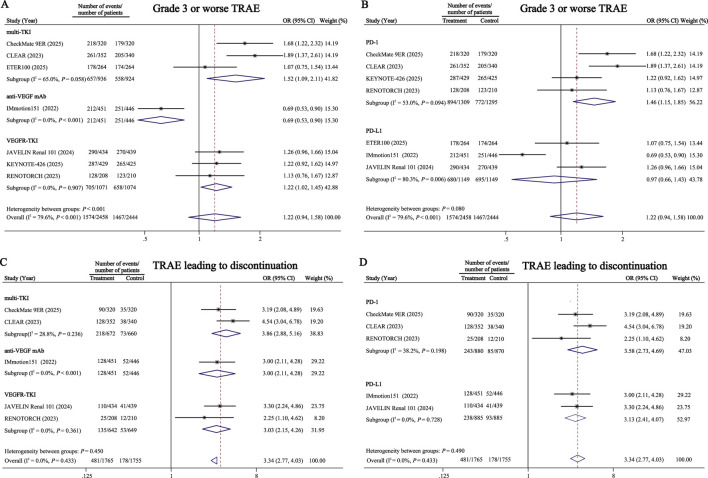
Subgroup analysis of Grade ≥3 TRAEs and discontinuation due to TRAEs by **(A,C)** anti-angiogenic drug type and **(B,D)** ICI type in advanced RCC. Abbreviations: TRAEs, treatment-related adverse events; ICI, immune checkpoint inhibitor; OR, odds ratio; CI, confidence interval; RCC, renal cell carcinoma.

Subgroup analyses were performed to investigate the observed heterogeneity and to characterize risks according to drug class. As shown in Figure 6, when stratified by the type of ICIs, no meaningful distinction was observed between the two ICI classes (PD-1 vs. PD-L1) regimens for either grade ≥3 TRAEs (*P* for interaction = 0.080) or TRAEs leading to discontinuation (*P* for interaction = 0.490). The point estimates for PD-1 inhibitors were 1.46 (95% CI: 1.15–1.85) for grade ≥3 TRAEs and 3.58 (95% CI: 2.73–4.69) for discontinuation, while for PD-L1 inhibitors they were 0.97 (95% CI: 0.66–1.43) and 3.13 (95% CI: 2.41–4.07), respectively.

Stratification by the type of anti-angiogenic drug showed distinct profiles in pairwise comparisons. For grade ≥3 TRAEs, anti-VEGF mAbs carried a significantly lower risk than multi-targeted TKIs (*P* for interaction <0.001). Notably, the risk associated with VEGFR-TKIs (1.22 [95% CI: 1.02–1.45]) was not significantly different from that of multi-TKIs (*P* for interaction = 0.244). At the within-class level, heterogeneity was completely eliminated in the VEGFR-TKI subgroup (I^2^ = 0.0%) and was absent by definition in the single-trial anti-VEGF mAb subgroup (I^2^ = 0.0%). Within the multi-TKI subgroup, moderate residual heterogeneity persisted (I^2^ = 65.0%), driven by divergent grade ≥3 TRAE estimates across cabozantinib (OR = 1.68), lenvatinib (OR = 1.89), and anlotinib-based regimens (OR = 1.07). For TRAEs leading to discontinuation, no statistically meaningful differences were found among the three subgroups. The risk estimates were comparable for anti-VEGF mAbs (3.00 [95% CI: 2.11–4.28]), multi-TKIs (3.86 [95% CI: 2.88–5.16]), and VEGFR-TKIs (3.03 [95% CI: 2.15–4.26]).

The spectrum of specific grade ≥3 toxicities is detailed in Table 2. Combination therapy significantly increased the odds of weight loss, increased ALT, increased AST, hypertension, hyponatremia, proteinuria, and rash. Conversely, classic sunitinib-associated hematologic toxicities including anemia, thrombocytopenia, and neutropenia were significantly less frequent with combination therapy. Fatigue was also reduced. Subgroup analysis of these specific toxicities by drug class (Supplementary Table S4) revealed distinct profiles. The risk of diarrhea was significantly elevated with PD-1-based regimens and VEGFR-TKIs, but not with PD-L1 inhibitors or anti-VEGF mAbs. Hypertension risk was consistently elevated across most subgroups but showed significant interaction by VEGF inhibitor class (*P* for interaction = 0.028), being most pronounced with multi-TKIs. The protective effect against hematologic toxicities was consistent across all subgroups.

**TABLE 2:** Incidence of grade ≥3 TRAEs in patients with advanced RCC treated with first-line ICIs plus anti-angiogenic agents versus sunitinib.

Safety outcome	No. of trials	OR (95% CI)	*P*	I^2^ (%)
Diarrhea	6	1.56 (0.90, 2.69)	0.112	70.6
Decreased appetite	6	1.59 (0.96, 2.64)	0.069	45.5
Weight loss	3	16.10 (3.87, 66.94)*	<0.001	45.1
Vomiting	6	0.66 (0.33, 1.30)	0.230	41.8
Palmar-plantar erythrodysesthesia syndrome	6	1.13 (0.66, 1.93)	0.647	63.6
ALT increased	4	4.04 (2.66, 6.12)	<0.001	20.1
AST increased	4	2.86 (1.77, 4.64)	<0.001	13.7
Hypertension	6	1.33 (1.01, 1.76)	0.043	70.8
Hyponatremia	2	2.22 (1.16, 4.26)	0.016	49.2
Proteinuria	5	1.93 (1.35, 2.76)	<0.001	43.6
Lipase increased	3	1.38 (0.88, 2.18)	0.162	0.0
Anemia	6	0.10 (0.05, 0.18)	<0.001	0.0
Thrombocytopenia	5	0.05 (0.02, 0.12)	<0.001	0.0
Neutropenia	5	0.07 (0.04, 0.14)	<0.001	0.7
Rash	5	3.85 (1.67, 8.87)	0.002	0.0
Fatigue	5	0.58 (0.41, 0.83)	0.002	18.0

Abbreviation: OR, odd ratios; ICIs, immune checkpoint inhibitors; TRAEs, treatment-related adverse events; CI, confidence interval; RCC, renal cell carcinoma.

* The pooled OR, for weight loss is based on extremely sparse control-arm events (2 events across 1,043 patients in the CLEAR, ETER100, and JAVELIN, Renal 101 trials).

### 3.5 Sensitivity analyses

Sensitivity analysis provided support for the consistency of the efficacy and safety outcomes. The complete results are provided in Supplementary Table S5. Regarding PFS, the sequential exclusion of individual trials yielded consistent hazard ratios ranging from 0.60 (95% CI: 0.53–0.67) to 0.66 (95% CI: 0.58–0.74), with statistical significance retained for every omitted study (*P* < 0.001). Likewise, OS estimates remained stable after removal of any single trial, with hazard ratios between 0.81 (95% CI: 0.74–0.88) and 0.84 (95% CI: 0.77–0.91), and significance was preserved (*P* < 0.001). Notably, when IMmotion151 was excluded, the pooled OS HR became 0.81 (95% CI: 0.74–0.88) with heterogeneity reduced to 0.0%. Grade ≥3 TRAE showed ORs from 1.13 (95% CI: 0.88–1.46) to 1.36 (95% CI: 1.19–1.54), with statistical significance achieved upon exclusion of IMmotion151 (*P* < 0.001).

To evaluate whether geographic and ethnic patient composition contributed to the observed heterogeneity, a sensitivity analysis was performed restricting the analysis to the five multinational trials (excluding ETER100 and RENOTORCH, which enrolled exclusively Chinese patients). I^2^ values in this restricted analysis were 80.6% for PFS, 91.6% for ORR, 81.7% for DCR, and 86.2% for grade ≥3 TRAEs.

### 3.6 Analysis of publication bias

Funnel plots and Egger’s regression test results for each outcome are shown in Supplementary Figures S4,S5, respectively. Visual inspection of the funnel plots along with statistical testing revealed the absence of significant publication bias for several key outcomes. Specifically, PFS (*P* = 0.283), ORR (*P* = 0.131), DCR (*P* = 0.166), grade ≥3 TRAEs (*P* = 0.404), and TRAEs leading to discontinuation (*P* = 0.448) all demonstrated displayed a symmetric distribution of effect estimates, with the corresponding statistical tests yielding non-significant *P*-values.

In contrast, significant funnel plot asymmetry was observed for OS (*P* = 0.004). To evaluate the stability of the summary estimates and account for potential missing studies, the non-parametric trim-and-fill analysis was applied. For OS, the method imputed three hypothetical studies to achieve symmetry, yielding an adjusted hazard ratio of 0.86 (95% CI: 0.80–0.92, *P* < 0.001). A sensitivity analysis excluding the two studies reporting interim results (ETER100 and RENOTORCH) was performed for OS. In this reduced set of five studies, Egger’s test yielded a non-significant result (*P* = 0.186).

## 4 Discussion

This meta-analysis demonstrates that combining ICIs plus anti-angiogenic agents offers superior clinical outcomes compared with sunitinib monotherapy as first-line treatment for advanced or metastatic RCC. The pooled analysis demonstrated a 37% reduction in the risk of disease progression and a 17% reduction in the risk of death. Survival benefits were most marked in poor- and intermediate-risk patients, whereas favorable-risk patients showed no significant OS gain. Notably, the class of anti-angiogenic agents significantly influenced both efficacy and tolerability. Collectively, these findings establish ICI-based combination regimens as a new standard of care, with treatment selection guided by IMDC risk group, anti-angiogenic agent class, and individual tolerability.

The observed clinical benefits are underpinned by robust biological rationale. Approximately 80% of RCC cases are clear cell histology, which harbors biallelic inactivation of the von Hippel-Lindau tumor suppressor gene. This inactivation leads to constitutive stabilization of hypoxia-inducible factors (HIF). The stabilization of HIF results in subsequent transcriptional activation of VEGF, platelet-derived growth factor, and other pro-angiogenic mediators (Clark, 2009; Mazumder et al., 2023; Shen and Kaelin, 2013). However, VEGF signaling extends beyond vascular effects to profoundly modulate the tumor immune microenvironment. Elevated VEGF levels facilitate the recruitment and activation of immunosuppressive cell populations, such as myeloid-derived suppressor cells, regulatory T cells, and M2 macrophages, and at the same time suppress key functions of antigen-presenting cells and T-cell infiltration into the tumor parenchyma (Kim et al., 2022; Zhang and Brekken, 2022). Normalization of the aberrant tumor vasculature by anti-angiogenic agents improves tissue oxygenation and reduces hypoxia-driven immunosuppression. Inhibition of VEGFR signaling decreases the recruitment of MDSCs and Tregs. Restoration of endothelial adhesion molecule expression facilitates T-cell extravasation (Lee et al., 2020). When combined with PD-1/PD-L1 blockade that reinvigorates exhausted tumor-infiltrating lymphocytes, this remodeled microenvironment enables a more effective immune response (Lee et al., 2020).

Our subgroup analyses stratified by IMDC risk criteria revealed a gradient in therapeutic benefit. The most pronounced benefit was observed in poor-risk patients, who experienced a 54% decrease in the risk of disease progression and a 48% decrease in the risk of mortality. Intermediate-risk patients, who account for the largest proportion of the advanced RCC population, also experienced substantial and clinically relevant benefits for both survival endpoints (Jena, 2020). In contrast, favorable-risk patients demonstrated a statistically significant PFS improvement yet failed to demonstrate a statistically significant OS advantage. From a heterogeneity perspective, IMDC-based stratification serves a dual and mechanistically distinct role. For PFS and OS, it functions as a genuine biological effect modifier, with stratification markedly reducing within-subgroup PFS heterogeneity and virtually eliminating OS heterogeneity across all strata (Supplementary Table S3). This pattern reflects the true differential magnitude of survival benefit across prognostic strata and represents a clinically actionable signal. For ORR, by contrast, IMDC-based stratification did not resolve heterogeneity, as substantial within-subgroup I^2^ persisted in the intermediate-risk and poor-risk subgroups, driven in each case by the markedly higher estimate from ETER100. This divergence is itself informative, indicating that the dominant source of ORR variability lies outside IMDC risk composition and instead arises from drug class differences, particularly anlotinib’s distinct pharmacological profile, as further developed below. Consistent with this interpretation, all patient-level interaction tests in Supplementary Table S3 were non-significant, and the geographic sensitivity analysis restricted to multinational trials did not reduce I^2^ for any outcome. Together, these findings argue against patient-related factors as independent drivers of between-study variability.

Patients with the worst prognosis by IMDC risk classification derived the greatest relative benefit, which is biologically plausible. Poor-risk RCC is characterized by high tumor burden, elevated inflammatory markers, anemia, and thrombocytosis. These features reflect an aggressive tumor biology with high proliferative activity and angiogenic drive, which manifests clinically as rapid disease kinetics (Goksen and Arslan, 2025; Hamieh et al., 2017). Owing to their high vascularity and elevated VEGF expression, these tumors are heavily reliant on angiogenic signaling and may therefore be especially responsive to anti-angiogenic therapy (Rini, 2007). Poor-risk tumors tend to harbor high tumor mutational burden and a pronounced inflammatory milieu, generating abundant neoantigens available for T-cell recognition. Conversely, favorable-risk RCC is characterized by indolent disease behavior, lower tumor burden, and longer intervals from diagnosis to systemic therapy (Di Civita et al., 2026). The absence of OS benefit in favorable-risk patients raises the question of whether sequential monotherapy may suffice, sparing unnecessary toxicity.

Subgroup analyses stratified by anti-angiogenic drug class revealed an efficacy gradient that offers practical guidance for treatment selection. The test for interaction across drug classes was statistically significant for PFS, confirming genuine differences in treatment effect magnitude. The greatest PFS risk reduction was observed with multi-targeted TKI-based regimens, followed by VEGFR-selective TKI-based regimens, with the anti-VEGF mAb-based regimen showing the most attenuated benefit. Class-based stratification markedly reduced within-subgroup PFS heterogeneity, confirming that anti-angiogenic drug class is the primary structural driver of PFS variability between trials.

The efficacy gradient across drug classes is mechanistically grounded. Multi-targeted TKIs inhibit not only VEGFR1-3 but also additional oncogenic kinases including MET, AXL, RET, KIT, and FLT3 (Osanto and van der Hulle, 2018; Song et al., 2020; Cabanillas and Habra, 2016). This broader kinase inhibitory footprint, particularly MET and AXL blockade, suppresses key immunosuppressive signaling pathways and may synergize more potently with PD-1/PD-L1 blockade than selective VEGFR inhibition alone. VEGFR-selective TKIs achieved intermediate and consistent benefit, reflecting the efficacy of targeted VEGFR blockade without the additional oncogenic pathway inhibition of multi-targeted agents. IMmotion151 emerged as a consistent outlier across all efficacy endpoints. Bevacizumab specifically targets VEGF-A without inhibiting receptor-level or intracellular kinase signaling, mechanistically precluding the direct anti-tumor cytotoxicity that underpins the superior PFS of TKI-based regimens.

For OS, the bevacizumab-based combination failed to demonstrate a statistically significant survival advantage, in contrast to the significant benefits achieved by both TKI-based subclasses. Sensitivity analysis confirmed IMmotion151’s singular influence on OS heterogeneity, with its exclusion reducing I^2^ to 0.0%. The PFS-to-OS disconnect within IMmotion151 is consistent with its pharmacological profile, as bevacizumab’s selective VEGF-A neutralization achieves transient vascular normalization but lacks the direct anti-tumor cytotoxicity required to produce a durable survival advantage over sunitinib.

However, while the OS evidence robustly distinguishes TKI-based from bevacizumab-based regimens, it does not produce a proportional gradient within the TKI class that mirrors the PFS hierarchy. Two complementary confounds explain this attenuation. First, systematic asymmetry in subsequent ICI exposure dilutes between-arm OS differences. Sunitinib-treated patients, accessing ICI for the first time after progression, received subsequent ICI-based therapy at rates 14 to 53 percentage points higher than their combination-arm counterparts, effectively compressing the OS gap that first-line efficacy differences would otherwise produce. The CLEAR trial illustrates this mechanism: despite a statistically significant HR of 0.79, the median OS values were numerically similar (53.7 vs. 54.3 months), because the substantial asymmetry in subsequent ICI exposure (54.6% vs. 15.8%) compressed the between-arm gap during peak progression events and caused the median to fall within the zone of maximal curve convergence. This demonstrates that under substantial post-progression crossover, the HR is the more appropriate interpretive metric, while median OS systematically underestimates the true survival advantage of the more efficacious first-line regimen. Second, first-line treatment intensity and post-progression option breadth may be inversely related. Patients progressing on multi-TKI combinations may face a narrowed subsequent TKI landscape due to prior broad-spectrum kinase inhibitor exposure, whereas those progressing on VEGFR-selective TKI or bevacizumab-based regimens retain wider access to effective subsequent therapies. This inference is directionally consistent with patterns in Supplementary Table S6 but cannot be formally quantified and should be interpreted as a mechanistically plausible contributor rather than an established confound.

Beyond these trial-level confounds, OS is structurally less suited than PFS to discriminate between first-line regimens in this evidence base. OS integrates cumulative multi-line treatment effects rather than capturing first-line efficacy alone, and OS data maturity varied substantially across trials, with ETER100 and RENOTORCH providing only interim data at median follow-up of 22.8 and 14.6 months respectively. Third, the asymmetric post-progression ICI exposure documented in Supplementary Table S6 generates non-proportional hazards that render median OS a misleading summary statistic and further attenuate the discriminatory power of OS between first-line regimens. Accordingly, the absence of a statistically significant OS gradient across TKI subclasses reflects the structural insensitivity of OS in this specific evidence base, not evidence of equivalent long-term survival benefit. TKI-based combinations are robustly preferred over bevacizumab-based regimens on OS grounds; within the TKI class, PFS and ORR remain the most pharmacologically interpretable endpoints for guiding drug class selection.

The tumor response outcomes provide the most granular evidence regarding heterogeneity sources. For ORR, class-based stratification revealed a two-tiered heterogeneity structure. At the between-class level, IMmotion151 diverged markedly from all other regimens, with the only non-significant ORR estimate spanning the null. The between-class interaction was highly significant, and exclusion of IMmotion151 produced the largest single-study reduction in overall ORR heterogeneity, confirming drug class as the dominant source of ORR variability. At the within-class level, heterogeneity was substantially reduced in the VEGFR-TKI subgroup but remained high within the multi-TKI subgroup, reflecting pharmacological and contextual differences among individual agents. The ORR gradient across cabozantinib, lenvatinib, and anlotinib likely reflects distinct but complementary immunomodulatory mechanisms. Cabozantinib’s MET and AXL inhibition reverses HGF-induced PD-L1 upregulation, suppresses MDSC accumulation, and expands intratumoral CD8^+^ T-cell infiltration, establishing an immune-permissive microenvironment not achievable through VEGFR blockade alone (Bergerot et al., 2019). Lenvatinib’s FGFR1-4 inhibition reactivates tumor-cell IFNγ signaling and reduces immunosuppressive TAM infiltration, conferring superior anti-PD-1 synergy over VEGFR-selective agents in preclinical models (Adachi et al., 2022). Anlotinib’s immunomodulatory mechanism operates primarily at the vascular level, downregulating endothelial cell PD-L1 via AKT inactivation and thereby facilitating CD8^+^ T-cell transmigration (Liu et al., 2020). ETER100’s interim data nature, as discussed in the OS analysis above, may additionally inflate the ORR point estimate through inclusion of unconfirmed or maturing responses. These pharmacological and methodological differences co-occur within ETER100 and cannot be formally decomposed at the study level.

The DCR results present a contrasting heterogeneity pattern. Class-based stratification completely resolved within-subgroup DCR heterogeneity across all three drug class subgroups, whereas ORR heterogeneity persisted within the multi-TKI subgroup even after stratification. This divergence indicates that disease stabilization is pharmacologically class-determined and within-class consistent, while objective tumor shrinkage is more sensitive to individual agent differences within the multi-TKI class. The practical implication is that the choice of specific multi-TKI agent may more substantially influence the probability of achieving an objective response than of achieving disease control, a consideration of particular relevance when rapid tumor shrinkage is a therapeutic priority.

Subgroup analysis by ICI type indicated that both anti-PD-1 and anti-PD-L1 antibodies conferred significant survival benefits, with no meaningful difference between them. This equivalence is biologically plausible given that both drug classes target the same immunosuppressive axis (Beckermann et al., 2017; Hamanishi et al., 2016). Therefore, treatment selection can be guided primarily by the anti-angiogenic agent and its specific efficacy-safety profile rather than the particular ICI employed. Across both CPS and IC scoring methodologies, PD-L1 expression status did not significantly modify the treatment benefit of combination therapy for either PFS or OS. This is mechanistically consistent with the known capacity of anti-angiogenic agents to remodel the immunosuppressive tumor microenvironment independently of baseline PD-L1 status, thereby potentially rendering ICI efficacy less dependent on pre-existing PD-L1 expression than is observed with ICI monotherapy (Zhou et al., 2024). Accordingly, PD-L1 expression should not be used as a criterion to select or exclude patients from first-line ICI-based combination therapy in advanced RCC.

The safety analysis revealed that the pooled odds of grade ≥3 TRAEs were higher but did not reach statistical significance, whereas the incidence of TRAEs leading to treatment discontinuation in the combination therapy group was more than three times that of the control group. Subgroup analyses by anti-angiogenic drug class revealed safety differences that mirror the two-tiered structure observed for efficacy. IMmotion151 was the only regimen showing a significantly reduced grade ≥3 TRAE risk relative to sunitinib, consistent with bevacizumab’s absence of direct kinase inhibition. Multi-TKI combinations carried a significantly elevated grade ≥3 TRAE burden, with moderate residual within-class heterogeneity driven by the divergent safety profile of anlotinib (ETER100). VEGFR-TKI combinations showed a consistent and predictable safety profile with no within-class heterogeneity. Anlotinib’s lower relative TRAE burden compared with cabozantinib and lenvatinib likely reflects two factors: a kinase inhibitory spectrum that lacks the potent MET and AXL blockade contributing to cabozantinib’s off-target toxicity, and an intermittent dosing schedule (2-week-on/1-week-off) that reduces cumulative kinase exposure relative to the continuous daily dosing of both cabozantinib and lenvatinib. Whether this lower TRAE burden reflects a genuine pharmacological advantage or a dosing-schedule artifact with attendant efficacy implications requires prospective head-to-head evaluation.

In contrast to the drug class variation observed for grade ≥3 TRAEs, the risk of treatment-limiting toxicity was uniformly elevated across all combinations. Complete absence of heterogeneity was observed both overall and within all drug class and ICI type subgroups for TRAEs leading to discontinuation, with an approximately 3-fold increase relative to sunitinib. This establishes treatment-limiting toxicity as a universal class effect of ICI-anti-angiogenic therapy, mandating proactive toxicity monitoring, structured patient counseling, and dose modification strategies regardless of which specific combination is employed.

Analysis of specific grade ≥3 toxicities revealed distinct patterns reflecting the pharmacological profiles of the combined agents. Hepatotoxicity was significantly increased with combination therapy. The negligible heterogeneity indicates consistent hepatotoxicity risk across all combinations. Subgroup analysis further indicated that TKI-based combinations were associated with higher hepatotoxicity rates (Supplementary Table S4). In patients receiving ICI plus TKI combinations, grade ≥3 transaminase elevation may reflect direct TKI-mediated hepatocellular injury, ICI-induced immune-mediated hepatitis, or a synergistic effect of both mechanisms, and management should be individualized accordingly. TKI-induced hepatotoxicity is typically managed through dose reduction or temporary interruption, whereas immune-mediated hepatitis requires prompt corticosteroid administration and, in severe cases, permanent ICI discontinuation (Brahmer et al., 2018; Haanen et al., 2022; Shah et al., 2013). Baseline liver function assessment and regular transaminase monitoring, particularly during the first 12 weeks, are essential across all regimens, with heightened vigilance for TKI-based combinations (Remash et al., 2021). Weight loss was dramatically increased with combination therapy; however, the pooled OR of 16.10 (95% CI: 3.87–66.94) must be interpreted with considerable caution. This estimate rests on only three trials with extremely sparse control-arm events (2 events across 1,043 patients), as CLEAR contributed negligible weight owing to zero events in its sunitinib arm, leaving the pooled result entirely dependent on ETER100 and JAVELIN Renal 101, which produced OR estimates diverging approximately 4.6-fold. Whether this signal reflects a genuine pharmacological effect or an artifact of sparse event reporting remains uncertain, and standardized body weight assessment in future RCTs is warranted. Hyponatremia showed a 2.2-fold increase, likely reflecting the combined effects of VEGF pathway inhibition on renal sodium handling and immune-mediated adrenal or thyroid dysfunction. Hypertension was modestly elevated with substantial heterogeneity reflecting differences across anti-angiogenic agents. Subgroup analysis by anti-angiogenic class revealed a significant interaction in hypertension risk, with multi-TKI combinations showing the highest risk and VEGFR-TKI combinations demonstrating an intermediate level of risk. By contrast, the anti-VEGF monoclonal antibody combination showed no significant increase in hypertension risk compared to sunitinib. This gradient aligns with the broader kinase inhibition spectrum of multi-targeted agents (Shyam et al., 2023). Proteinuria was significantly increased, consistent with the known on-target effect of VEGFR/VEGF blockade on the glomerular endothelium (Shyam et al., 2023). Diarrhea was significantly increased, although substantial heterogeneity was present. The elevated diarrhea risk was driven by TKI-containing combinations, whereas the bevacizumab-based combination actually showed a reduced diarrhea risk compared to sunitinib.

A striking and clinically meaningful finding was the dramatic reduction in hematologic toxicities with combination therapy compared to sunitinib monotherapy. Anemia was reduced by 90%, thrombocytopenia by 95%, and neutropenia by 93%. The biological explanation lies in the profound myelosuppressive effects of sunitinib, which inhibits multiple kinases involved in hematopoiesis including colony-stimulating factor 1 receptor, c-KIT and FLT3 (Galanis and Levis, 2015; van Erp et al., 2010). While TKI components of combination regimens also inhibit some of these kinases, the typical dosing strategies adopted in such combinations often employ lower TKI doses to accommodate the partnering agent. This dose reduction consequently results in diminished myelosuppression compared to full-dose TKI monotherapy. The anti-VEGF mAb combination (IMmotion151) showed particularly pronounced reductions in hematologic toxicity, consistent with the absence of direct kinase inhibition by bevacizumab. Additionally, fatigue was significantly reduced with combination therapy.

Sequential omission of individual studies supported the stability of the efficacy and safety results. Excluding IMmotion151 was particularly informative, as it reduced OS heterogeneity to 0.0% and shifted the grade ≥3 TRAE pooled OR to statistical significance, confirming IMmotion151 as the single most influential source of both OS and safety heterogeneity. A complementary geographic sensitivity analysis restricted to the five multinational trials yielded I^2^ values unchanged or marginally higher than the full-set analysis, providing direct evidence that geographic patient composition does not independently drive between-study variability. ETER100’s outlier behavior reflects the pharmacological characteristics of anlotinib and its interim data nature rather than population differences. Together with the subgroup analyses, these sensitivity analyses reveal two mechanistically distinct and complementary sources of heterogeneity. The first is anti-angiogenic drug class, principally the contrast between bevacizumab’s selective VEGF-A neutralization and TKI-based broader kinase inhibition, and secondarily individual agent differences within the multi-TKI class. This factor accounts for the dominant heterogeneity in ORR, DCR, and grade ≥3 TRAEs. The second is IMDC risk category as a biological effect modifier for survival outcomes, with stratification substantially reducing PFS heterogeneity and virtually eliminating OS heterogeneity. These two sources operate through fundamentally different mechanisms and are not competing explanations. Drug class heterogeneity is resolved by class-stratified analyses, while IMDC-driven heterogeneity is leveraged for individualized patient selection. Together they define the two axes of the clinical decision framework discussed below.

Assessment of publication bias yielded reassuring results for most outcomes but identified potential concerns for two endpoints. Significant funnel plot asymmetry was detected for OS. After trim-and-fill correction, the OS effect size remained statistically robust and clinically meaningful. Accordingly, while the primary pooled OS estimate (HR 0.83) may be marginally overestimated due to this asymmetry, the adjusted estimate (HR 0.86, 95% CI: 0.80–0.92) remains statistically significant and clinically meaningful, and the overall conclusion of a significant OS benefit is preserved even under this conservative correction. This indicates that even under a conservative worst-case scenario, the survival benefit associated with the intervention is preserved. Crucially, a targeted sensitivity analysis offers a more mechanistic explanation for the observed asymmetry in the OS funnel plot. Two of the included trials (ETER100 and RENOTORCH) contributed data derived from interim analyses rather than final study reports (Yan et al., 2024; Zhou et al., 2025). Interim data are inherently characterized by immature event counts and evolving follow-up. Upon exclusion of these two studies, the remaining five-study dataset yielded a non-significant Egger’s test, no longer meeting the threshold for asymmetry.

The convergent findings from heterogeneity analyses, IMDC risk stratification, and drug class comparisons support a two-dimensional, patient-centered treatment selection framework (Table 3). It should be noted that the two dimensions were evaluated as independent subgroup analyses, as limited trial-level data and insufficient statistical power precluded formal two-dimensional interaction testing. The framework therefore represents a clinically reasoned synthesis rather than a statistically derived model.

**TABLE 3:** Two-dimensional patient-centered treatment-selection framework for first-line ICI plus anti-angiogenic therapy in advanced RCC.

Clinical setting	Priority: rapid tumor reduction (high symptom burden, visceral crisis, rapidly progressive disease)	Priority: efficacy-toxicity balance (routine first-line)	Combination therapy contraindicated or patient-declined
Poor/intermediate risk (significant OS and PFS benefit)	Multi-targeted TKI + ICI^a^ Greatest PFS and ORR benefit; highest grade ≥3 TRAE burden; within-class ORR variability exists, and individual agent selection may influence response probability	VEGFR-selective TKI + ICI^a^ Significant PFS and OS benefit; consistent and predictable safety profile; balanced first-line option	Sunitinib monotherapy^b^
Favorable risk (significant PFS benefit; OS benefit not demonstrated)	Multi-targeted TKI + ICI^c^ Significant PFS benefit; no OS advantage; highest toxicity burden relative to uncertain survival gain; individualized risk-benefit discussion is therefore required	VEGFR-selective TKI + ICI^c^ Significant PFS benefit; no OS advantage; balanced tolerability; individualized risk-benefit discussion required	Sunitinib monotherapy^b^
Universal toxicity monitoring (all combination regimens)	Prior to initiation, counsel all patients that treatment discontinuation risk is markedly elevated versus sunitinib, a universal class effect independent of regimen, and that structured dose-modification protocols are required Throughout treatment: monitor hepatic function, blood pressure, and urinary protein regardless of anti-angiogenic agent class. ICI class (anti-PD-1 vs. anti-PD-L1) does not influence efficacy or safety and should not drive regimen selection		Sunitinib toxicity profile: anticipate elevated rates of grade ≥3 hematologic toxicities (anemia, thrombocytopenia, neutropenia) and fatigue; standard TKI monitoring applies

Recommendation strength:

^a^ Strongly recommended: significant OS and PFS benefit, well-characterized safety profile.

^b^ Standard of care when combination therapy is contraindicated or declined: avoids the markedly elevated discontinuation risk of combination regimens; sequential therapy upon progression remains viable.

^c^ Recommended with individualized discussion: significant PFS benefit; OS advantage absent or uncertain.

Abbreviations: ICI, immune checkpoint inhibitor; TKI, tyrosine kinase inhibitor; VEGFR, vascular endothelial growth factor receptor; mAb, monoclonal antibody; OS, overall survival; PFS, progression-free survival; RCC, renal cell carcinoma.

The first dimension is IMDC risk stratification, which determines the strength of the recommendation for combination therapy. Poor- and intermediate-risk patients derive clear, statistically significant OS benefits and represent the population for whom ICI-anti-angiogenic combination therapy is most strongly indicated, regardless of which TKI-based regimen is chosen. Favorable-risk patients demonstrate significant PFS benefit but no significant OS advantage. Given the approximately 3-fold increased treatment discontinuation risk established as a universal class effect, individualized risk-benefit discussion is warranted in this subgroup, and sequential monotherapy strategies warrant prospective investigation.

The second dimension is anti-angiogenic drug class selection. The framework encompasses TKI-based ICI combinations only, as the anti-VEGF mAb-based regimen (atezolizumab plus bevacizumab) failed to demonstrate significant OS or ORR benefits in the pooled analysis. Sunitinib monotherapy accordingly remains the appropriate standard of care when ICI-containing combination therapy is contraindicated or declined. When rapid tumor shrinkage is critical, multi-TKI combinations offer the highest ORR and greatest PFS risk reduction, at the cost of the highest grade ≥3 TRAE burden. When a balance of efficacy and tolerability is preferred, VEGFR-TKI combinations provide intermediate PFS benefit with a consistent and predictable safety profile. Across all regimens, the approximately 3-fold elevated discontinuation risk constitutes a universal class effect, mandating proactive patient counseling, dose-modification protocols, and systematic monitoring of hepatic function, blood pressure, and proteinuria prior to and throughout treatment.

Several limitations must be considered. First, all seven trials are open-label with respect to participant and personnel blinding, rated as unclear risk of bias for this domain across all studies. While open-label design is practically unavoidable for regimens with distinct toxicity profiles, it carries differential implications across endpoints. OS and PFS are determined by independently adjudicated events and are less susceptible to detection bias. In contrast, ORR and DCR rely on investigator-assessed tumor measurements, and knowledge of treatment assignment may influence the categorization of borderline responses, potentially inflating response estimates in the combination arms. Second, study-level meta-analyses are not amenable to adjustment for patient-level prognostic factors or unmeasured confounders. Third, the varying lengths of follow-up across trials, with some studies (ETER100, RENOTORCH) reporting interim analyses with immature OS data, introduces uncertainty in the precision of survival estimates. Fourth, post-progression treatment access was heterogeneous across trials, with subsequent ICI use in control arms ranging from 23.2% to 81.0%, producing a functionally crossover-like survival confound. Combined with the inherent integration of cumulative multi-line treatment effects in OS, this structurally reduces the sensitivity of OS to between-regimen differences that manifest primarily during the first-line period.

Future research priorities should address several critical knowledge gaps identified by our analysis. First, an individual patient data meta-analysis would enable adjusted subgroup comparisons and exploration of treatment-by-biomarker interactions. Second, prospective validation of tumor mutational burden, gene expression signatures, circulating tumor DNA, and immune cell infiltration is needed for precision patient selection. Third, comparative effectiveness studies directly comparing different ICI-TKI combinations in head-to-head randomized trials would inform treatment selection more definitively than the indirect comparisons possible from our network of trials. Fourth, exploring novel combinations with emerging targets such as LAG-3, TIM-3, TIGIT, and HIF-2α inhibitors could further improve outcomes, especially in patients resistant to current regimens.

## 5 Conclusion

This meta-analysis shows that first-line ICIs plus anti-angiogenic agents significantly improve PFS, OS, ORR, and DCR versus sunitinib in advanced RCC. Survival benefits were most pronounced in poor-risk and intermediate-risk patients, whereas favorable-risk patients showed no significant OS gain. The class of anti-angiogenic agent was the primary determinant of both efficacy and tolerability: multi-targeted TKIs offered the greatest tumor control at the cost of higher toxicity, VEGFR-selective TKIs provided a balanced efficacy-safety profile, and anti-VEGF mAb-based combinations failed to demonstrate significant OS or ORR advantages. ICI class did not influence outcomes and should not drive regimen selection. Although combination therapy increased the risk of hepatotoxicity, and treatment discontinuation, these were partially counterbalanced by markedly lower rates of hematologic toxicities and fatigue compared with sunitinib. These findings support a two-dimensional clinical decision framework in which IMDC risk stratification guides the strength of the recommendation for combination therapy and anti-angiogenic agent class is matched to individual clinical priorities and tolerability.

## Data Availability

The original contributions presented in the study are included in the article/Supplementary Material, further inquiries can be directed to the corresponding author.

## Supplementary material

SUPPLEMENTARY FIGURE S1 Forest plots of **(A)** ORR and **(B)** DCR for ICIs plus anti-angiogenic therapy versus sunitinib in first-line treatment of advanced RCC. Abbreviations: ICIs, immune checkpoint inhibitors; ORR, objective response rate; DCR, disease control rate; OR, odds ratio; CI, confidence interval; RCC, renal cell carcinoma.

SUPPLEMENTARY FIGURE S2 Forest plot of subgroup analysis for ORR based on IMDC risk levels in advanced RCC. Abbreviations: ORR, objective response rate; IMDC, International Metastatic RCC Database Consortium; RCC, renal cell carcinoma; OR, odds ratio; CI, confidence interval.

SUPPLEMENTARY FIGURE S3 Forest plots of subgroup analyses for **(A,C)** anti-angiogenic drug types and **(B,D)** ICI types on ORR and DCR in advanced RCC. Abbreviations: ORR, objective response rate; DCR, disease control rate; ICI, immune checkpoint inhibitor; RCC, renal cell carcinoma; OR, odds ratio; CI, confidence interval.

SUPPLEMENTARY FIGURE S4 Funnel plots of publication bias. Abbreviation: PFS, progression free survival; OS, overall survival; ORR, objective response rate; DCR, disease control rate; TRAEs, treatment-related adverse events; HR, hazard ratio; OR, odd ratios.

SUPPLEMENTARY FIGURE S5 Egger's test of publication bias. Abbreviation: PFS, progression free survival; OS, overall survival; ORR, objective response rate; DCR, disease control rate; TRAEs, treatment-related adverse events; HR, hazard ratio; OR, odd ratios.
