# Supplementary material for: First-line immune checkpoint inhibitor plus anti-angiogenic therapy for advanced renal cell carcinoma: a meta-analysis of randomized controlled trials
Source: Front Pharmacol. 2026 Jul 27;17:1850724. doi: 10.3389/fphar.2026.1850724 (PMC13454646; doi:10.3389/fphar.2026.1850724)

Study (Year)	Number of events/number of patients: Treatment	Control	OR (95% CI)	Weight (%)
Favorable				
CheckMate 9ER (2025)	49/74	31/72	2.59 (1.33, 5.07)	6.44
ETER100 (2025)	30/37	17/36	4.79 (1.67, 13.70)	3.73
JAVELIN Renal 101 (2024)	71/94	44/96	3.65 (1.97, 6.77)	6.96
Subgroup (I^2 = 0.0%, P = 0.583)	150/205	92/204	3.34 (2.20, 5.06)	17.14
Intermediate				
CheckMate 9ER (2025)	105/188	52/188	3.31 (2.15, 5.09)	9.12
ETER100 (2025)	136/188	46/189	8.13 (5.13, 12.89)	8.75
JAVELIN Renal 101 (2024)	163/270	87/277	3.33 (2.34, 4.73)	10.09
RENOTORCH (2023)	95/169	56/174	2.71 (1.74, 4.20)	9.00
Subgroup (I^2 = 78.1%, P = 0.003)	499/815	241/828	3.91 (2.50, 6.12)	36.97
Poor				
CheckMate 9ER (2025)	26/61	7/68	6.47 (2.55, 16.45)	4.40
ETER100 (2025)	23/39	3/38	16.77 (4.39, 64.09)	2.58
JAVELIN Renal 101 (2024)	28/73	11/71	3.39 (1.53, 7.53)	5.34
RENOTORCH (2023)	24/41	9/37	4.39 (1.66, 11.64)	4.14
Subgroup (I^2 = 31.1%, P = 0.226)	101/214	30/214	5.52 (3.06, 9.96)	16.46
Intermediate/Poor				
CheckMate 9ER (2025)	131/249	59/256	3.71 (2.53, 5.44)	9.71
ETER100 (2025)	159/227	49/227	8.49 (5.55, 12.99)	9.19
JAVELIN Renal 101 (2024)	191/343	98/348	3.21 (2.34, 4.40)	10.53
Subgroup (I^2 = 85.4%, P = 0.001)	481/819	206/831	4.60 (2.61, 8.08)	29.43
Heterogeneity between groups: P = 0.551				
Overall (I^2 = 63.3%, P < 0.001)	1231/2053	569/2077	4.23 (3.32, 5.38)	100.00

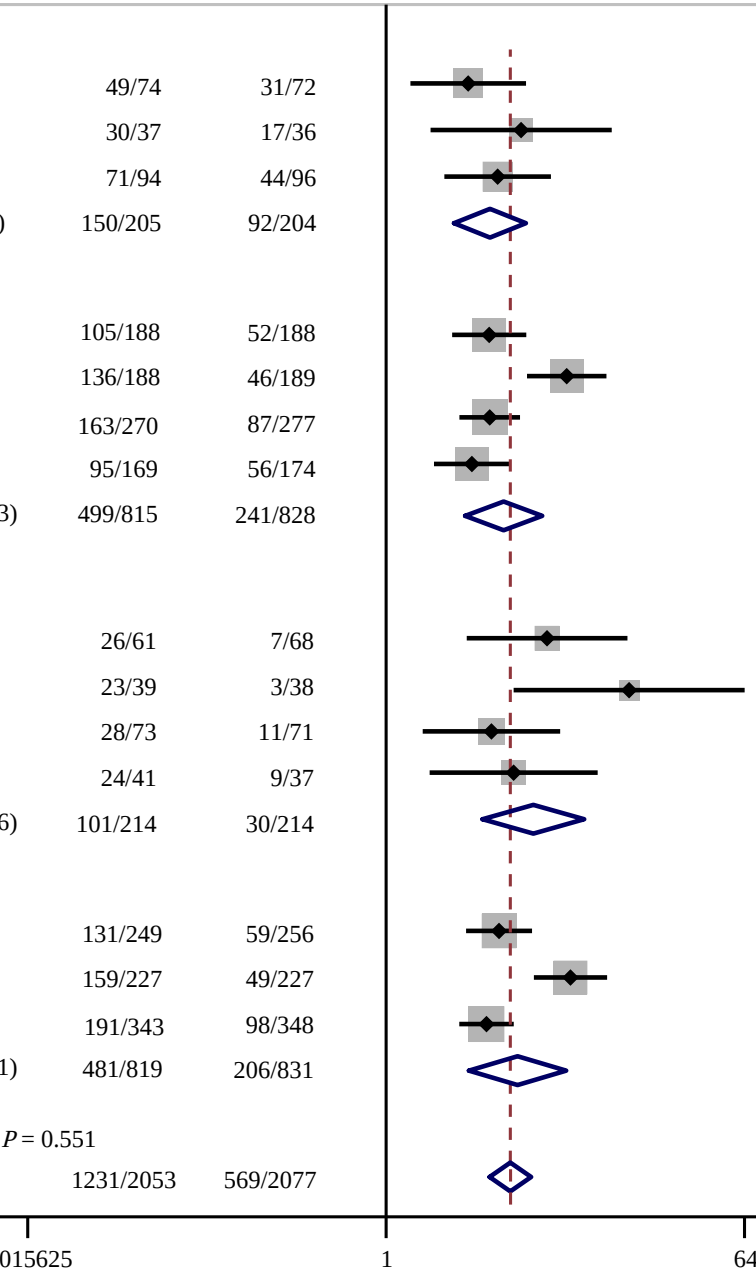

Supplement: Supplementary file 2 [file DataSheet2.pdf]
