# Supplementary material for: First-line immune checkpoint inhibitor plus anti-angiogenic therapy for advanced renal cell carcinoma: a meta-analysis of randomized controlled trials
Source: Front Pharmacol. 2026 Jul 27;17:1850724. doi: 10.3389/fphar.2026.1850724 (PMC13454646; doi:10.3389/fphar.2026.1850724)
Supplement: Supplementary file 3 [file DataSheet3.pdf]

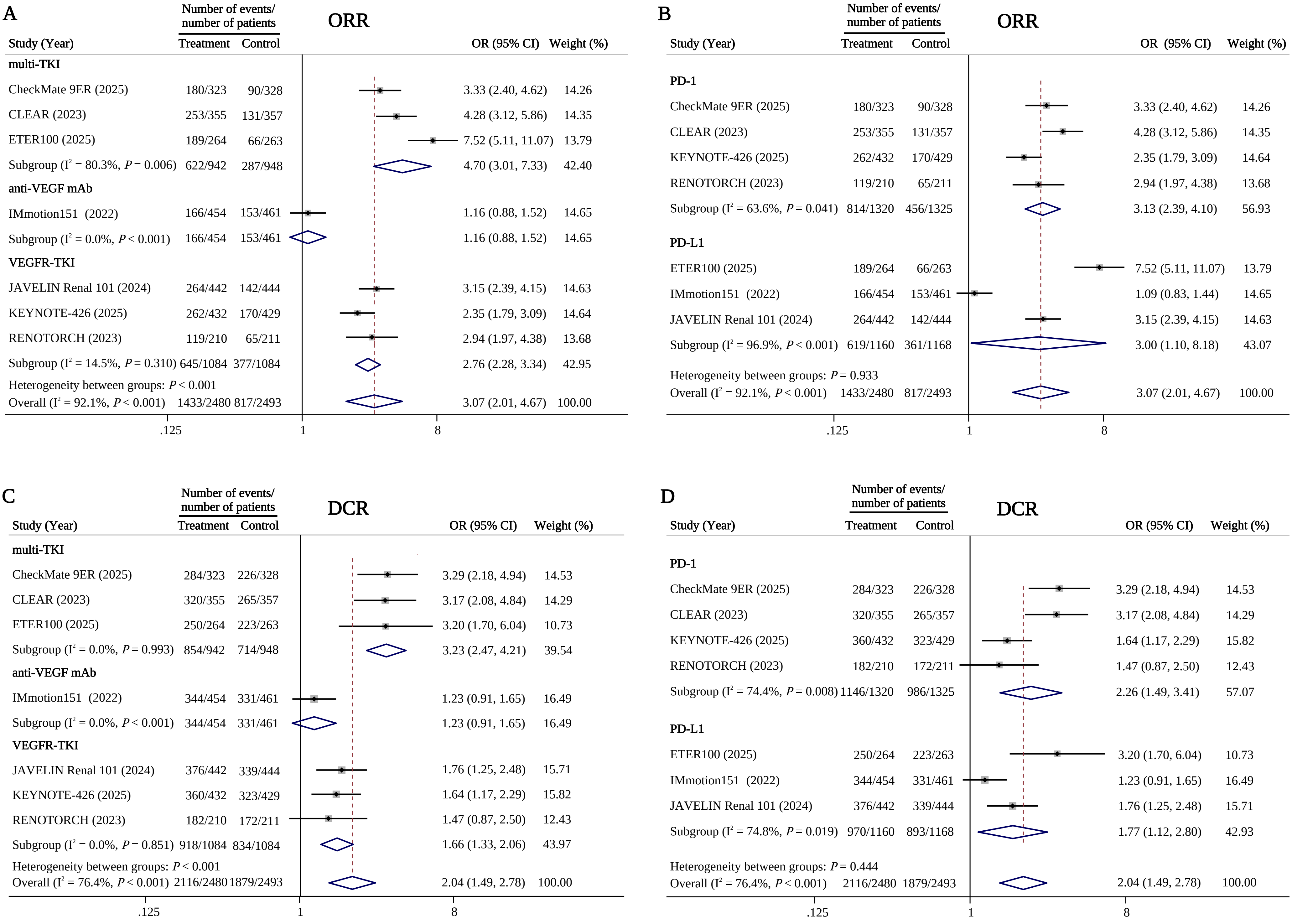
A
Number of events/ number of patients
ORR
Study (Year) Treatment Control OR (95% CI) Weight (%)
multi-TKI
CheckMate 9ER (2025) 180/323 90/328 3.33 (2.40, 4.62) 14.26
CLEAR (2023) 253/355 131/357 4.28 (3.12, 5.86) 14.35
ETER100 (2025) 189/264 66/263 7.52 (5.11, 11.07) 13.79
Subgroup (I² = 80.3%, P = 0.006) 622/942 287/948 4.70 (3.01, 7.33) 42.40
anti-VEGF mAb
IMmotion151 (2022) 166/454 153/461 1.16 (0.88, 1.52) 14.65
Subgroup (I² = 0.0%, P < 0.001) 166/454 153/461 1.16 (0.88, 1.52) 14.65
VEGFR-TKI
JAVELIN Renal 101 (2024) 264/442 142/444 3.15 (2.39, 4.15) 14.63
KEYNOTE-426 (2025) 262/432 170/429 2.35 (1.79, 3.09) 14.64
RENOTORCH (2023) 119/210 65/211 2.94 (1.97, 4.38) 13.68
Subgroup (I² = 14.5%, P = 0.310) 645/1084 377/1084 2.76 (2.28, 3.34) 42.95
Heterogeneity between groups: P < 0.001
Overall (I² = 92.1%, P < 0.001) 1433/2480 817/2493 3.07 (2.01, 4.67) 100.00
.125
1
8
B
Number of events/ number of patients
ORR
Study (Year) Treatment Control OR (95% CI) Weight (%)
PD-1
CheckMate 9ER (2025) 180/323 90/328 3.33 (2.40, 4.62) 14.26
CLEAR (2023) 253/355 131/357 4.28 (3.12, 5.86) 14.35
KEYNOTE-426 (2025) 262/432 170/429 2.35 (1.79, 3.09) 14.64
RENOTORCH (2023) 119/210 65/211 2.94 (1.97, 4.38) 13.68
Subgroup (I² = 63.6%, P = 0.041) 814/1320 456/1325 3.13 (2.39, 4.10) 56.93
PD-L1
ETER100 (2025) 189/264 66/263 7.52 (5.11, 11.07) 13.79
IMmotion151 (2022) 166/454 153/461 1.09 (0.83, 1.44) 14.65
JAVELIN Renal 101 (2024) 264/442 142/444 3.15 (2.39, 4.15) 14.63
Subgroup (I² = 96.9%, P < 0.001) 619/1160 361/1168 3.00 (1.10, 8.18) 43.07
Heterogeneity between groups: P = 0.933
Overall (I² = 92.1%, P < 0.001) 1433/2480 817/2493 3.07 (2.01, 4.67) 100.00
.125
1
8
C
Number of events/ number of patients
DCR
Study (Year) Treatment Control OR (95% CI) Weight (%)
multi-TKI
CheckMate 9ER (2025) 284/323 226/328 3.29 (2.18, 4.94) 14.53
CLEAR (2023) 320/355 265/357 3.17 (2.08, 4.84) 14.29
ETER100 (2025) 250/264 223/263 3.20 (1.70, 6.04) 10.73
Subgroup (I² = 0.0%, P = 0.993) 854/942 714/948 3.23 (2.47, 4.21) 39.54
anti-VEGF mAb
IMmotion151 (2022) 344/454 331/461 1.23 (0.91, 1.65) 16.49
Subgroup (I² = 0.0%, P < 0.001) 344/454 331/461 1.23 (0.91, 1.65) 16.49
VEGFR-TKI
JAVELIN Renal 101 (2024) 376/442 339/444 1.76 (1.25, 2.48) 15.71
KEYNOTE-426 (2025) 360/432 323/429 1.64 (1.17, 2.29) 15.82
RENOTORCH (2023) 182/210 172/211 1.47 (0.87, 2.50) 12.43
Subgroup (I² = 0.0%, P = 0.851) 918/1084 834/1084 1.66 (1.33, 2.06) 43.97
Heterogeneity between groups: P < 0.001
Overall (I² = 76.4%, P < 0.001) 2116/2480 1879/2493 2.04 (1.49, 2.78) 100.00
.125
1
8
D
Number of events/ number of patients
DCR
Study (Year) Treatment Control OR (95% CI) Weight (%)
PD-1
CheckMate 9ER (2025) 284/323 226/328 3.29 (2.18, 4.94) 14.53
CLEAR (2023) 320/355 265/357 3.17 (2.08, 4.84) 14.29
KEYNOTE-426 (2025) 360/432 323/429 1.64 (1.17, 2.29) 15.82
RENOTORCH (2023) 182/210 172/211 1.47 (0.87, 2.50) 12.43
Subgroup (I² = 74.4%, P = 0.008) 1146/1320 986/1325 2.26 (1.49, 3.41) 57.07
PD-L1
ETER100 (2025) 250/264 223/263 3.20 (1.70, 6.04) 10.73
IMmotion151 (2022) 344/454 331/461 1.23 (0.91, 1.65) 16.49
JAVELIN Renal 101 (2024) 376/442 339/444 1.76 (1.25, 2.48) 15.71
Subgroup (I² = 74.8%, P = 0.019) 970/1160 893/1168 1.77 (1.12, 2.80) 42.93
Heterogeneity between groups: P = 0.444
Overall (I² = 76.4%, P < 0.001) 2116/2480 1879/2493 2.04 (1.49, 2.78) 100.00
.125
1
8
