# Supplementary material for: First-line immune checkpoint inhibitor plus anti-angiogenic therapy for advanced renal cell carcinoma: a meta-analysis of randomized controlled trials
Source: Front Pharmacol. 2026 Jul 27;17:1850724. doi: 10.3389/fphar.2026.1850724 (PMC13454646; doi:10.3389/fphar.2026.1850724)
Supplement: Supplementary file 4 [file DataSheet4.pdf]

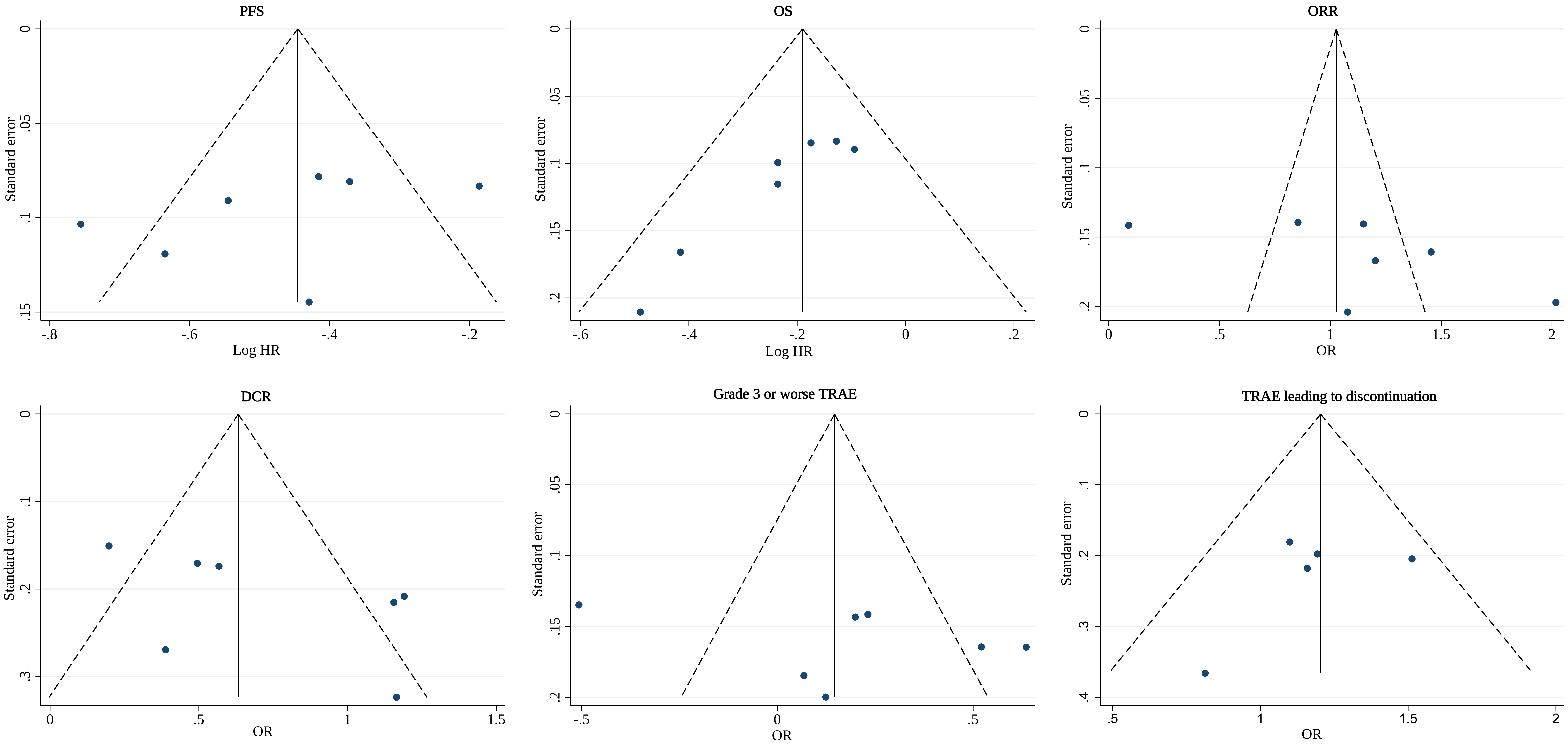

PFS
Standard error
0
.05
.1
.15
-.8
-.6
-.4
-.2
Log HR
OS
Standard error
0
.05
.1
.15
.2
-.6
-.4
-.2
0
.2
Log HR
ORR
Standard error
0
.05
.1
.15
.2
0
.5
1
1.5
2
OR
DCR
Standard error
0
.1
.2
.3
0
.5
1
1.5
OR
Grade 3 or worse TRAE
Standard error
0
.05
.1
.15
.2
-.5
0
.5
OR
TRAE leading to discontinuation
Standard error
0
.1
.2
.3
.4
.5
1
1.5
2
OR
