# Supplementary material for: First-line immune checkpoint inhibitor plus anti-angiogenic therapy for advanced renal cell carcinoma: a meta-analysis of randomized controlled trials
Source: Front Pharmacol. 2026 Jul 27;17:1850724. doi: 10.3389/fphar.2026.1850724 (PMC13454646; doi:10.3389/fphar.2026.1850724)
Supplement: Supplementary file 5 [file DataSheet5.pdf]

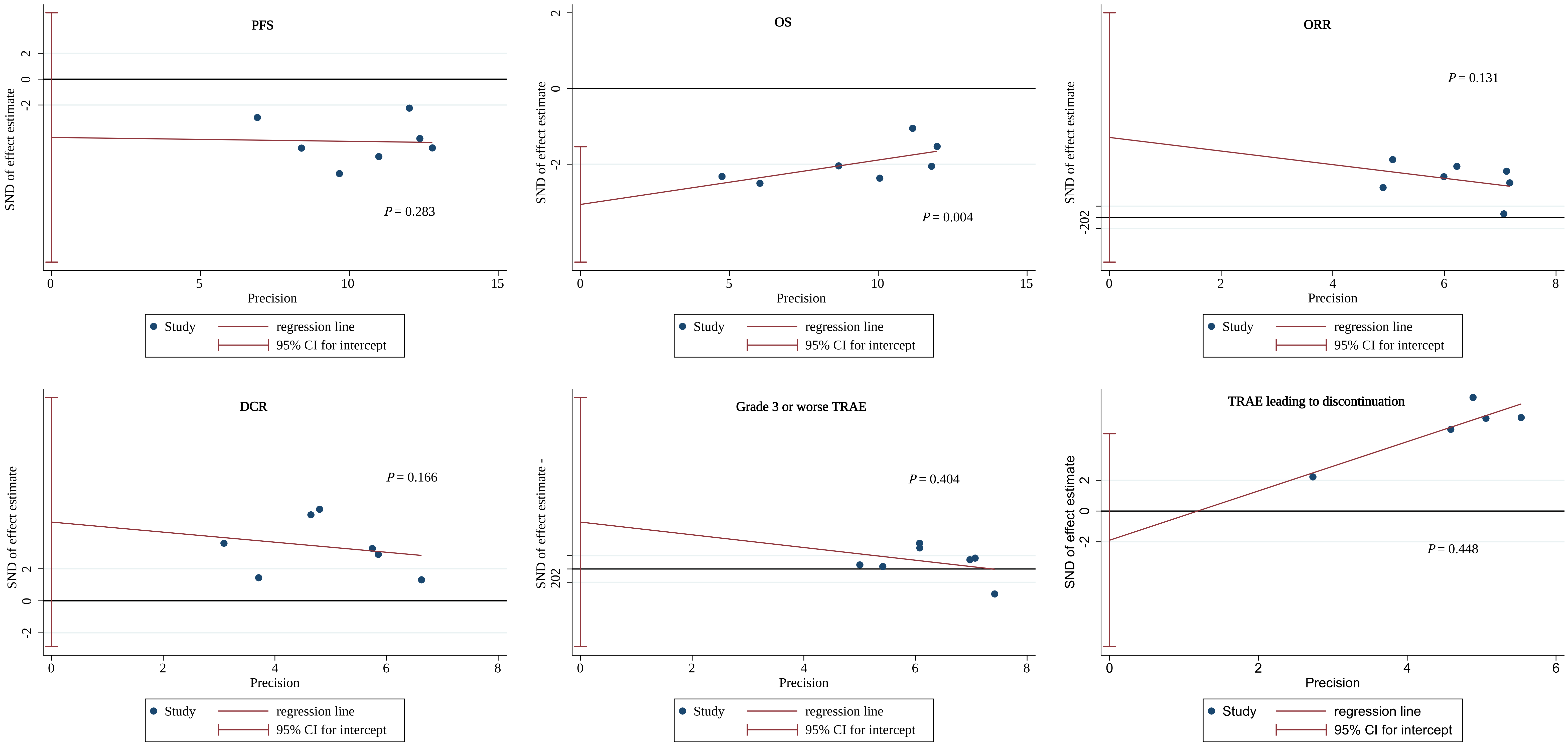
PFS
SND of effect estimate
Precision
P = 0.283
Study
regression line
95% CI for intercept
OS
SND of effect estimate
Precision
P = 0.004
Study
regression line
95% CI for intercept
ORR
SND of effect estimate
Precision
P = 0.131
Study
regression line
95% CI for intercept
DCR
SND of effect estimate
Precision
P = 0.166
Study
regression line
95% CI for intercept
Grade 3 or worse TRAE
SND of effect estimate
Precision
P = 0.404
Study
regression line
95% CI for intercept
TRAE leading to discontinuation
SND of effect estimate
Precision
P = 0.448
Study
regression line
95% CI for intercept
