# Supplementary material for: First-line immune checkpoint inhibitor plus anti-angiogenic therapy for advanced renal cell carcinoma: a meta-analysis of randomized controlled trials
Source: Front Pharmacol. 2026 Jul 27;17:1850724. doi: 10.3389/fphar.2026.1850724 (PMC13454646; doi:10.3389/fphar.2026.1850724)
Supplement: Supplementary file 6 [file Table1.docx]

**Supplementary Table 1.** literature-search methods

| Database | Search terms |
| --- | --- |
| Pubmed | #1 (((((((((((((((((((((((((((((((((((((((((((((("Carcinoma, Renal Cell"[Mesh]) OR (Carcinomas, Renal Cell[Title/Abstract])) OR (Renal Cell Carcinomas[Title/Abstract])) OR (Renal Carcinoma[Title/Abstract])) OR (Carcinoma, Renal[Title/Abstract])) OR (Renal Carcinomas[Title/Abstract])) OR (Nephroid Carcinoma[Title/Abstract])) OR (Carcinoma, Nephroid[Title/Abstract])) OR (Nephroid Carcinomas[Title/Abstract])) OR (Adenocarcinoma, Renal Cell[Title/Abstract])) OR (Adenocarcinomas, Renal Cell[Title/Abstract])) OR (Renal Cell Adenocarcinoma[Title/Abstract])) OR (Renal Cell Adenocarcinomas[Title/Abstract])) OR (Renal Cell Carcinoma[Title/Abstract])) OR (Renal Cell Cancer[Title/Abstract])) OR (Cancer, Renal Cell[Title/Abstract])) OR (Renal Cell Cancers[Title/Abstract])) OR (Adenocarcinoma, Renal[Title/Abstract])) OR (Renal Adenocarcinoma[Title/Abstract])) OR (Renal Adenocarcinomas[Title/Abstract])) OR (Adenocarcinoma Of Kidney[Title/Abstract])) OR (Adenocarcinoma Of Kidneys[Title/Abstract])) OR (Kidney, Adenocarcinoma Of[Title/Abstract])) OR (Clear Cell Renal Cell Carcinoma[Title/Abstract])) OR (Carcinoma, Hypernephroid[Title/Abstract])) OR (Hypernephroid Carcinoma[Title/Abstract])) OR (Hypernephroid Carcinomas[Title/Abstract])) OR (Grawitz Tumor[Title/Abstract])) OR (Tumor, Grawitz[Title/Abstract])) OR (Hypernephroma[Title/Abstract])) OR (Hypernephromas[Title/Abstract])) OR (Clear Cell Renal Carcinoma[Title/Abstract])) OR (Papillary Renal Cell Carcinoma[Title/Abstract])) OR (Chromophil Renal Cell Carcinoma[Title/Abstract])) OR (Renal Cell Carcinoma, Papillary[Title/Abstract])) OR (Chromophobe Renal Cell Carcinoma[Title/Abstract])) OR (Collecting Duct Carcinoma (Kidney[Title/Abstract]))) OR (Carcinoma, Collecting Duct (Kidney[Title/Abstract]))) OR (Carcinomas, Collecting Duct (Kidney[Title/Abstract]))) OR (Collecting Duct Carcinomas (Kidney[Title/Abstract]))) OR (Collecting Duct Carcinoma[Title/Abstract])) OR (Carcinoma, Collecting Duct[Title/Abstract])) OR (Carcinomas, Collecting Duct[Title/Abstract])) OR (Collecting Duct Carcinomas[Title/Abstract])) OR (Collecting Duct Carcinoma of the Kidney[Title/Abstract])) OR (Renal Collecting Duct Carcinoma[Title/Abstract])) OR (Sarcomatoid Renal Cell Carcinoma[Title/Abstract])  #2 ((((((((((((((((((((((("Antibodies, Monoclonal, Humanized"[Mesh]) OR (Antibodies, Humanized[Title/Abstract])) OR (Humanized Antibodies[Title/Abstract])) OR (nivolumab[Title/Abstract])) OR (pembrolizumab[Title/Abstract])) OR (cemiplimab[Title/Abstract])) OR (dostarlimab[Title/Abstract])) OR (retifanlimab[Title/Abstract])) OR (toripalimab[Title/Abstract])) OR (sintilimab[Title/Abstract])) OR (camrelizumab[Title/Abstract])) OR (tislelizumab[Title/Abstract])) OR (serplulimab[Title/Abstract])) OR (penpulimab[Title/Abstract])) OR (atezolizumab[Title/Abstract])) OR (durvalumab[Title/Abstract])) OR (avelumab[Title/Abstract])) OR (envafolimab[Title/Abstract])) OR (socazolimab[Title/Abstract])) OR (sugemalimab[Title/Abstract])) OR (ipilimumab[Title/Abstract])) OR (tremelimumab[Title/Abstract])) OR (quavonlimab[Title/Abstract])) OR ((((((((((((((((((((((((((((((((("Sorafenib"[Mesh]) OR (BAY 43-9006[Title/Abstract])) OR (BAY 439006[Title/Abstract])) OR (BAY 43 9006[Title/Abstract])) OR (Sorafenib Tosylate[Title/Abstract])) OR (4-(4-(3-(4-Chloro-3-trifluoromethylphenyl)ureido)phenoxy)pyridine-2-carboxylic acid methyamide-4-methylbenzenesulfonate[Title/Abstract])) OR (Nexavar[Title/Abstract])) OR (BAY 545-9085[Title/Abstract])) OR (BAY 5459085[Title/Abstract])) OR (BAY 545 9085[Title/Abstract])) OR (BAY-545-9085[Title/Abstract])) OR (BAY5459085[Title/Abstract])) OR (Sorafenib N-Oxide[Title/Abstract])) OR (Sorafenib N Oxide[Title/Abstract])) OR (BAY-673472[Title/Abstract])) OR (BAY 673472[Title/Abstract])) OR (bevacizumab[Title/Abstract])) OR (ramucirumab[Title/Abstract])) OR (IBI305[Title/Abstract])) OR (lenvatinib[Title/Abstract])) OR (cabozantinib[Title/Abstract])) OR (regorafenib[Title/Abstract])) OR (rivoceranib[Title/Abstract])) OR (anlotinib[Title/Abstract])) OR (apatinib[Title/Abstract])) OR (fruquintinib[Title/Abstract])) OR (famitinib[Title/Abstract])) OR (donafenib[Title/Abstract])) OR (sunitinib[Title/Abstract])) OR (pazopanib[Title/Abstract])) OR (axitinib[Title/Abstract])) OR (vandetanib[Title/Abstract])) OR (nintedanib[Title/Abstract])) OR ((((((((((((((((((((((((((((("Antineoplastic Combined Chemotherapy Protocols"[Mesh]) OR (Combined Antineoplastic Agents[Title/Abstract])) OR (Antineoplastic Agents, Combined[Title/Abstract])) OR (Agent, Combined Antineoplastic[Title/Abstract])) OR (Agents, Combined Antineoplastic[Title/Abstract])) OR (Antineoplastic Agent, Combined[Title/Abstract])) OR (Combined Antineoplastic Agent[Title/Abstract])) OR (Anticancer Drug Combinations[Title/Abstract])) OR (Anticancer Drug Combination[Title/Abstract])) OR (Drug Combination, Anticancer[Title/Abstract])) OR (Drug Combinations, Anticancer[Title/Abstract])) OR (Drug Combinations, Antineoplastic[Title/Abstract])) OR (Antineoplastic Combined Chemotherapy Regimens[Title/Abstract])) OR (Antineoplastic Drug Combinations[Title/Abstract])) OR (Antineoplastic Drug Combination[Title/Abstract])) OR (Combinations, Antineoplastic Drug[Title/Abstract])) OR (Drug Combination, Antineoplastic[Title/Abstract])) OR (Antineoplastic Chemotherapy Protocols[Title/Abstract])) OR (Antineoplastic Chemotherapy Protocol[Title/Abstract])) OR (Chemotherapy Protocol, Antineoplastic[Title/Abstract])) OR (Protocol, Antineoplastic Chemotherapy[Title/Abstract])) OR (Protocols, Antineoplastic Chemotherapy[Title/Abstract])) OR (Cancer Chemotherapy Protocols[Title/Abstract])) OR (Cancer Chemotherapy Protocol[Title/Abstract])) OR (Chemotherapy Protocol, Cancer[Title/Abstract])) OR (Chemotherapy Protocols, Cancer[Title/Abstract])) OR (Protocol, Cancer Chemotherapy[Title/Abstract])) OR (Protocols, Cancer Chemotherapy[Title/Abstract])) OR (Chemotherapy Protocols, Antineoplastic[Title/Abstract]))  #3 randomized controlled trial[Publication Type] OR  randomized[Title/Abstract] OR  placebo[Title/Abstract]  #4 #1 AND #2 AND #3 |
| Embase | #1 'antibodies, monoclonal, humanized'/exp  #2 'antibodies, and humanized':ab,ti  #3 'humanized antibodies':ab,ti  #4 'nivolumab':ab,ti  #5 'pembrolizumab':ab,ti  #6 'cemiplimab':ab,ti  #7 'dostarlimab':ab,ti  #8 'retifanlimab':ab,ti  #9 'toripalimab':ab,ti  #10 'sintilimab':ab,ti  #11 'camrelizumab':ab,ti  #12 'tislelizumab':ab,ti  #13 'serplulimab':ab,ti  #14 'penpulimab':ab,ti  #15 'atezolizumab':ab,ti  #16 'durvalumab':ab,ti  #17 'avelumab':ab,ti  #18 'envafolimab':ab,ti  #19 'socazolimab':ab,ti  #20 'sugemalimab':ab,ti  #21 'ipilimumab':ab,ti  #22 'tremelimumab':ab,ti  #23 'quavonlimab':ab,ti  #24 'sorafenib'/exp  #25 'bay 43-9006':ab,ti  #26 'bay 43 9006':ab,ti  #27 'sorafenib tosylate':ab,ti  #28 '4-(4-(3-(4-chloro-3-trifluoromethylphenyl)ureido)phenoxy)pyridine-2-carboxylic acid methyamide-4-methylbenzenesulfonate':ab,ti  #29 'nexavar':ab,ti  #30 'bay 545-9085':ab,ti  #31 'bay 5459085':ab,ti  #32 'bay 545 9085':ab,ti  #33 'bay-545-9085':ab,ti  #34 'bay5459085':ab,ti  #35 'sorafenib n-oxide':ab,ti  #36 'sorafenib n oxide':ab,ti  #37 'bay-673472':ab,ti  #38 'bay 673472':ab,ti  #39 'bevacizumab':ab,ti  #40 'ramucirumab':ab,ti  #41 'IBI305':ab,ti  #42 'sorafenib':ab,ti  #43 'lenvatinib':ab,ti  #44 'cabozantinib':ab,ti  #45 'regorafenib':ab,ti  #46 'rivoceranib':ab,ti  #47 'anlotinib':ab,ti  #48 'apatinib':ab,ti  #49 'fruquintinib':ab,ti  #50 'famitinib':ab,ti  #51 'donafenib':ab,ti  #52 'sunitinib':ab,ti  #53 'pazopanib':ab,ti  #54 'axitinib':ab,ti  #55 'vandetanib':ab,ti  #56 'nintedanib':ab,ti  #57 'antineoplastic combined chemotherapy protocols'/exp  #58 'combined antineoplastic agents':ab,ti  #59 'antineoplastic agents, combined':ab,ti  #60 'agent, combined antineoplastic':ab,ti  #61 'agents, combined antineoplastic':ab,ti  #62 'antineoplastic agent, combined':ab,ti  #63 'combined antineoplastic agent':ab,ti  #64 'anticancer drug combinations':ab,ti  #65 'anticancer drug combination':ab,ti  #66 'drug combination, anticancer':ab,ti  #67 'drug combinations, anticancer':ab,ti  #68 'drug combinations, antineoplastic':ab,ti  #69 'antineoplastic combined chemotherapy regimens':ab,ti  #70 'antineoplastic drug combinations':ab,ti  #71 'antineoplastic drug combination':ab,ti  #72 'combinations, antineoplastic drug':ab,ti  #73 'drug combination, antineoplastic':ab,ti  #74 'antineoplastic chemotherapy protocols':ab,ti  #75 'antineoplastic chemotherapy protocol':ab,ti  #76 'chemotherapy protocol, antineoplastic':ab,ti  #77 'protocol, antineoplastic chemotherapy':ab,ti  #78 'protocols, antineoplastic chemotherapy':ab,ti  #79 'cancer chemotherapy protocols':ab,ti  #80 'cancer chemotherapy protocol':ab,ti  #81 'chemotherapy protocol, cancer':ab,ti  #82 'chemotherapy protocols, cancer':ab,ti  #83 'protocol, cancer chemotherapy':ab,ti  #84 'protocols, cancer chemotherapy':ab,ti  #85 'chemotherapy protocols, antineoplastic':ab,ti  #86 #1 OR #2 OR #3 OR #4 OR #5 OR #6 OR #7 OR #8 OR #9 OR #10 OR #11 OR #12 OR #13 OR #14 OR #15 OR #16 OR #17 OR #18 OR #19 OR #20 OR #21 OR #22 OR #23 OR #24 OR #25 OR #26 OR #27 OR #28 OR #29 OR #30 OR #31 OR #32 OR #33 OR #34 OR #35 OR #36 OR #37 OR #38 OR #39 OR #40 OR #41 OR #42 OR #43 OR #44 OR #45 OR #46 OR #47 OR #48 OR #49 OR #50 OR #51 OR #52 OR #53 OR #54 OR #55 OR #56 OR #57 OR #58 OR #59 OR #60 OR #61 OR #62 OR #63 OR #64 OR #65 OR #66 OR #67 OR #68 OR #69 OR #70 OR #71 OR #72 OR #73 OR #74 OR #75 OR #76 OR #77 OR #78 OR #79 OR #80 OR #81 OR #82 OR #83 OR #84 OR #85  #87 'Carcinoma, Renal Cell'/exp  #88 'Carcinomas, Renal Cell':ab,ti  #89 'Renal Cell Carcinomas':ab,ti  #90 'Renal Carcinoma':ab,ti  #91 'Carcinoma, Renal':ab,ti  #92 'Renal Carcinomas':ab,ti  #93 'Nephroid Carcinoma':ab,ti  #94 'Carcinoma, Nephroid':ab,ti  #95 'Nephroid Carcinomas':ab,ti  #96 'Adenocarcinoma, Renal Cell':ab,ti  #97 'Adenocarcinomas, Renal Cell':ab,ti  #98 'Renal Cell Adenocarcinoma':ab,ti  #99 'Renal Cell Adenocarcinomas':ab,ti  #100 'Renal Cell Carcinoma':ab,ti  #101 'Renal Cell Cancer':ab,ti  #102 'Cancer, Renal Cell':ab,ti  #103 'Renal Cell Cancers':ab,ti  #104 'Adenocarcinoma, Renal':ab,ti  #105 'Renal Adenocarcinoma':ab,ti  #106 'Renal Adenocarcinomas':ab,ti  #107 'Adenocarcinoma Of Kidney':ab,ti  #108 'Adenocarcinoma Of Kidneys':ab,ti  #109 'Kidney, Adenocarcinoma Of':ab,ti  #110 'Clear Cell Renal Cell Carcinoma':ab,ti  #111 'Carcinoma, Hypernephroid':ab,ti  #112 'Hypernephroid Carcinoma':ab,ti  #113 'Hypernephroid Carcinomas':ab,ti  #114 'Grawitz Tumor':ab,ti  #115 'Tumor, Grawitz':ab,ti  #116 'Hypernephroma':ab,ti  #117 'Hypernephromas':ab,ti  #118 'Clear Cell Renal Carcinoma':ab,ti  #119 'Papillary Renal Cell Carcinoma':ab,ti  #120 'Chromophil Renal Cell Carcinoma':ab,ti  #121 'Renal Cell Carcinoma, Papillary':ab,ti  #122 'Chromophobe Renal Cell Carcinoma':ab,ti  #123 'Collecting Duct Carcinoma (Kidney)':ab,ti  #124 'Carcinoma, Collecting Duct (Kidney)':ab,ti  #125 'Carcinomas, Collecting Duct (Kidney)':ab,ti  #126 'Collecting Duct Carcinomas (Kidney)':ab,ti  #127 'Collecting Duct Carcinoma':ab,ti  #128 'Carcinoma, Collecting Duct':ab,ti  #129 'Carcinomas, Collecting Duct':ab,ti  #130 'Collecting Duct Carcinomas':ab,ti  #131 'Collecting Duct Carcinoma of the Kidney':ab,ti  #132 'Renal Collecting Duct Carcinoma':ab,ti  #133 'Sarcomatoid Renal Cell Carcinoma':ab,ti  #134 #87 OR #88 OR #89 OR #90 OR #91 OR #92 OR #93 OR #94 OR #95 OR #96 OR #97 OR #98 OR #99 OR #100 OR #101 OR #102 OR #103 OR #104 OR #105 OR #106 OR #107 OR #108 OR #109 OR #110 OR #111 OR #112 OR #113 OR #114 OR #115 OR #116 OR #117 OR #118 OR #119 OR #120 OR #121 OR #122 OR #123 OR #124 OR #125 OR #126 OR #127 OR #128 OR #129 OR #130 OR #131 OR #132 OR #133  #135 #86 AND #134  #136 #135 AND [randomized controlled trial]/lim |
| Cochrane library | #1 MeSH descriptor: [Carcinoma, Renal Cell] explode all trees  #2 (Carcinomas, Renal Cell):ti,ab,kw OR (Renal Cell Carcinomas):ti,ab,kw OR (Renal Carcinoma):ti,ab,kw OR (Carcinoma, Renal):ti,ab,kw OR (Renal Carcinomas):ti,ab,kw  #3 (Nephroid Carcinoma):ti,ab,kw OR (Carcinoma, Nephroid):ti,ab,kw OR (Nephroid Carcinomas):ti,ab,kw OR (Adenocarcinoma, Renal Cell):ti,ab,kw OR (Adenocarcinomas, Renal Cell):ti,ab,kw  #4 (Renal Cell Adenocarcinoma):ti,ab,kw OR (Renal Cell Adenocarcinomas):ti,ab,kw OR (Renal Cell Carcinoma):ti,ab,kw OR (Renal Cell Cancer):ti,ab,kw OR (Cancer, Renal Cell):ti,ab,kw  #5 (Renal Cell Cancers):ti,ab,kw OR (Adenocarcinoma, Renal):ti,ab,kw OR (Renal Adenocarcinoma):ti,ab,kw  #6 (Renal Cell Cancers):ti,ab,kw OR (Adenocarcinoma, Renal):ti,ab,kw OR (Renal Adenocarcinoma):ti,ab,kw  #7 (Renal Adenocarcinomas):ti,ab,kw OR (Adenocarcinoma Of Kidney):ti,ab,kw OR (Adenocarcinoma Of Kidneys):ti,ab,kw  #8 (Kidney, Adenocarcinoma Of):ti,ab,kw OR (Clear Cell Renal Cell Carcinoma):ti,ab,kw OR (Carcinoma, Hypernephroid):ti,ab,kw  #9 (Hypernephroid Carcinoma):ti,ab,kw OR (Hypernephroid Carcinomas):ti,ab,kw OR (Grawitz Tumor):ti,ab,kw  #10 (Tumor, Grawitz):ti,ab,kw OR (Hypernephroma):ti,ab,kw OR (Hypernephromas):ti,ab,kw  #11 (Clear Cell Renal Carcinoma):ti,ab,kw OR (Papillary Renal Cell Carcinoma  ):ti,ab,kw OR (Chromophil Renal Cell Carcinoma):ti,ab,kw  #12 (Renal Cell Carcinoma, Papillary):ti,ab,kw OR (Chromophobe Renal Cell Carcinoma):ti,ab,kw OR (Collecting Duct Carcinoma (Kidney)):ti,ab,kw  #13 (Carcinoma, Collecting Duct (Kidney)):ti,ab,kw OR (Carcinomas, Collecting Duct (Kidney)):ti,ab,kw OR (Collecting Duct Carcinomas (Kidney)):ti,ab,kw  #14 (Collecting Duct Carcinoma):ti,ab,kw OR (Carcinoma, Collecting Duct):ti,ab,kw OR (Carcinomas, Collecting Duct):ti,ab,kw  #15 (Collecting Duct Carcinomas):ti,ab,kw OR (Collecting Duct Carcinoma of the Kidney):ti,ab,kw OR (Renal Collecting Duct Carcinoma):ti,ab,kw  #16 (Sarcomatoid Renal Cell Carcinoma):ti,ab,kw  #17 #1 OR #2 OR #3 OR #4 OR #5 OR #6 OR #7 OR #8 OR #9 OR #10 OR #11 OR #12 OR #13 OR #14 OR #15 OR #16  #18 MeSH descriptor: [Antibodies, Monoclonal, Humanized] explode all trees  #19 (Antibodies, Humanized):ti,ab,kw OR (Humanized Antibodies):ti,ab,kw  #20 (nivolumab):ti,ab,kw OR (pembrolizumab):ti,ab,kw OR (cemiplimab):ti,ab,kw OR (dostarlimab):ti,ab,kw OR (retifanlimab):ti,ab,kw  #21 (toripalimab):ti,ab,kw OR (sintilimab):ti,ab,kw OR (camrelizumab):ti,ab,kw OR (tislelizumab):ti,ab,kw OR (serplulimab):ti,ab,kw  #22 (penpulimab):ti,ab,kw OR (atezolizumab):ti,ab,kw OR (durvalumab):ti,ab,kw OR (avelumab):ti,ab,kw OR (envafolimab):ti,ab,kw  #23 (socazolimab):ti,ab,kw OR (sugemalimab):ti,ab,kw OR (ipilimumab):ti,ab,kw OR (tremelimumab):ti,ab,kw OR (quavonlimab):ti,ab,kw  #24 #18 OR #19 OR #20 OR #21 OR #22 OR #23  #25 MeSH descriptor: [Sorafenib] explode all trees  #26 (BAY 43-9006):ti,ab,kw OR (BAY 439006):ti,ab,kw OR (BAY 43 9006):ti,ab,kw OR (Sorafenib Tosylate):ti,ab,kw OR (4-(4-(3-(4-Chloro-3-trifluoromethylphenyl)ureido)phenoxy)pyridine-2-carboxylic acid methyamide-4-methylbenzenesulfonate):ti,ab,kw  #27 (Nexavar):ti,ab,kw OR (BAY 545-9085):ti,ab,kw OR (BAY 5459085):ti,ab,kw OR (BAY 545 9085):ti,ab,kw OR (BAY-545-9085):ti,ab,kw  #28 (BAY5459085):ti,ab,kw OR (Sorafenib N-Oxide):ti,ab,kw OR (Sorafenib N Oxide):ti,ab,kw OR (BAY-673472):ti,ab,kw OR (BAY 673472):ti,ab,kw  #29 (bevacizumab):ti,ab,kw OR (ramucirumab):ti,ab,kw OR (IBI305):ti,ab,kw OR (sorafenib):ti,ab,kw OR (lenvatinib):ti,ab,kw  #30 (cabozantinib):ti,ab,kw OR (regorafenib):ti,ab,kw OR (rivoceranib):ti,ab,kw OR (anlotinib):ti,ab,kw OR (apatinib):ti,ab,kw  #31 (fruquintinib):ti,ab,kw OR (famitinib):ti,ab,kw OR (donafenib):ti,ab,kw OR (sunitinib):ti,ab,kw OR (pazopanib):ti,ab,kw  #32 (axitinib):ti,ab,kw OR (vandetanib):ti,ab,kw OR (nintedanib):ti,ab,kw  #33 #25 OR #26 OR #27 OR #28 OR #29 OR #30 OR #31 OR #32  #34 MeSH descriptor: [Antineoplastic Combined Chemotherapy Protocols] explode all trees  #35 (Combined Antineoplastic Agents):ti,ab,kw OR (Antineoplastic Agents, Combined):ti,ab,kw OR (Agent, Combined Antineoplastic):ti,ab,kw OR (Agents, Combined Antineoplastic):ti,ab,kw OR (Antineoplastic Agent, Combined):ti,ab,kw  #36 (Combined Antineoplastic Agent):ti,ab,kw OR (Anticancer Drug Combinations):ti,ab,kw OR (Anticancer Drug Combination):ti,ab,kw OR (Drug Combination, Anticancer):ti,ab,kw OR (Drug Combinations, Anticancer):ti,ab,kw  #37 (Drug Combinations, Antineoplastic):ti,ab,kw OR (Antineoplastic Combined Chemotherapy Regimens):ti,ab,kw OR (Antineoplastic Drug Combinations):ti,ab,kw OR (Antineoplastic Drug Combination):ti,ab,kw OR (Combinations, Antineoplastic Drug):ti,ab,kw  #38 (Drug Combination, Antineoplastic):ti,ab,kw OR (Antineoplastic Chemotherapy Protocols):ti,ab,kw OR (Antineoplastic Chemotherapy Protocol):ti,ab,kw OR (Chemotherapy Protocol, Antineoplastic):ti,ab,kw OR (Protocol, Antineoplastic Chemotherapy):ti,ab,kw  #39 (Protocols, Antineoplastic Chemotherapy):ti,ab,kw OR (Cancer Chemotherapy Protocols):ti,ab,kw OR (Cancer Chemotherapy Protocol):ti,ab,kw OR (Chemotherapy Protocol, Cancer):ti,ab,kw OR (Chemotherapy Protocols, Cancer):ti,ab,kw  #40 (Protocol, Cancer Chemotherapy):ti,ab,kw OR (Protocols, Cancer Chemotherapy):ti,ab,kw OR (Chemotherapy Protocols, Antineoplastic):ti,ab,kw  #41 #34 OR #35 OR #36 OR #37 OR #38 OR #39 OR #40  #42 #24 OR #33 OR #41  #43 (randomized controlled trial):pt OR (randomized):ti,ab,kw OR (placebo):ti,ab,kw  #33 #17 AND #42 AND #43 |
