# Supplementary material for: First-line immune checkpoint inhibitor plus anti-angiogenic therapy for advanced renal cell carcinoma: a meta-analysis of randomized controlled trials
Source: Front Pharmacol. 2026 Jul 27;17:1850724. doi: 10.3389/fphar.2026.1850724 (PMC13454646; doi:10.3389/fphar.2026.1850724)
Supplement: Supplementary file 7 [file Table2.docx]

**Supplementary Table 2.** Quality assessment of the included randomized controlled trials

| Study | Year | Random sequence generation | Allocation concealment | Blinding of participants and personnel | Blinding of outcome assessment | Incomplete outcome data | Selective reporting | Other bias |
| --- | --- | --- | --- | --- | --- | --- | --- | --- |
| CheckMate 9ER | 2025 | low risk | low risk | Unclear(investigators and patients were unmasked to the treatment assignment) | low risk | low risk | low risk | low risk |
| CLEAR | 2024 | low risk | low risk | Unclear(investigators and patients were unmasked to the treatment assignment) | low risk | low risk | low risk | low risk |
| ETER100 | 2025 | low risk | low risk | Unclear(investigators and patients were unmasked to the treatment assignment) | low risk | low risk | low risk | low risk |
| IMmotion151 | 2022 | low risk | low risk | Unclear(investigators and patients were unmasked to the treatment assignment) | low risk | low risk | low risk | low risk |
| JAVELIN Renal 101 | 2024 | low risk | low risk | Unclear(investigators and patients were unmasked to the treatment assignment) | low risk | low risk | low risk | low risk |
| KEYNOTE-426 | 2025 | low risk | low risk | Unclear(investigators and patients were unmasked to the treatment assignment) | low risk | low risk | low risk | low risk |
| RENOTORCH | 2023 | low risk | low risk | Unclear(investigators and patients were unmasked to the treatment assignment) | low risk | low risk | low risk | low risk |
