# Supplementary material for: First-line immune checkpoint inhibitor plus anti-angiogenic therapy for advanced renal cell carcinoma: a meta-analysis of randomized controlled trials
Source: Front Pharmacol. 2026 Jul 27;17:1850724. doi: 10.3389/fphar.2026.1850724 (PMC13454646; doi:10.3389/fphar.2026.1850724)
Supplement: Supplementary file 8 [file Table3.docx]

**Supplementary Table 3.** Subgroup Analysis of PFS and OS for ICIs Combined with Anti‑angiogenic Agents Versus Sunitinib in First‑line Treatment of Advanced RCC

| **Variable** | **No. of Trials** | **PFS** | | | **P for interaction** | **No. of Trials** | **OS** | | | **P for interaction** |
| --- | --- | --- | --- | --- | --- | --- | --- | --- | --- | --- |
|  |  | **HR (95% CI)** | **I^2^ (%)** | ***P* Value** |  |  | **HR (95% CI)** | **I^2^ (%)** | ***P* Value** |  |
| Age |  |  |  |  | 0.394 |  |  |  |  | 0.247 |
| ≥ 65 years | 5 | 0.65 (0.56, 0.76) | 28.8 | <0.001 |  | 3 | 0.89 (0.76, 1.05) | 0.0 | 0.182 |  |
| < 65 years | 5 | 0.60 (0.53, 0.67) | 48.0 | <0.001 |  | 3 | 0.79 (0.69, 0.90) | 0.0 | 0.001 |  |
| Sex |  |  |  |  | 0.733 |  |  |  |  | 0.513 |
| Female | 5 | 0.62 (0.52, 0.75) | 15.6 | <0.001 |  | 3 | 0.80 (0.65, 0.98) | 65.6 | 0.029 |  |
| Male | 5 | 0.60 (0.54, 0.67) | 55.4 | <0.001 |  | 3 | 0.86 (0.76, 0.97) | 0.0 | 0.018 |  |
| ECOG score |  |  |  |  | 0.624 |  |  |  |  |  |
| 0 | 3 | 0.66 (0.54, 0.80) | 0.0 | <0.001 |  |  |  |  |  |  |
| 1 | 3 | 0.61 (0.51, 0.74) | 0.0 | <0.001 |  |  |  |  |  |  |
| Sarcomatoid Component |  |  |  |  | 0.314 |  |  |  |  |  |
| Yes | 2 | 0.35 (0.18, 0.69) | 0.0 | 0.002 |  |  |  |  |  |  |
| No | 2 | 0.50 (0.43, 0.59) | 42.4 | <0.001 |  |  |  |  |  |  |
| Previous nephrectomy |  |  |  |  | 0.147 |  |  |  |  |  |
| Yes | 3 | 0.60 (0.51, 0.71) | 0.0 | <0.001 |  |  |  |  |  |  |
| No | 3 | 0.75 (0.58, 0.95) | 0.0 | 0.020 |  |  |  |  |  |  |
| Liver metastasis |  |  |  |  | 0.825 |  |  |  |  |  |
| Yes | 3 | 0.56 (0.41, 0.77) | 0.0 | <0.001 |  |  |  |  |  |  |
| No | 2 | 0.59 (0.48, 0.72) | 0.0 | <0.001 |  |  |  |  |  |  |
| Bone metastasis |  |  |  |  | 0.379 |  |  |  |  |  |
| Yes | 3 | 0.50 (0.38, 0.67) | 11.2 | <0.001 |  |  |  |  |  |  |
| No | 2 | 0.59 (0.48, 0.72) | 0.0 | <0.001 |  |  |  |  |  |  |
| Lung metastasis |  |  |  |  | 0.569 |  |  |  |  |  |
| Yes | 3 | 0.59 (0.51, 0.68) | 0.0 | <0.001 |  |  |  |  |  |  |
| No | 2 | 0.53 (0.38, 0.74) | 0.0 | <0.001 |  |  |  |  |  |  |
| Number of metastatic organs or sites |  |  |  |  | 0.241 |  |  |  |  |  |
| 1 | 4 | 0.60 (0.49, 0.73) | 0.0 | <0.001 |  |  |  |  |  |  |
| ≥ 2 | 3 | 0.70 (0.60, 0.81) | 0.0 | <0.001 |  |  |  |  |  |  |
| PD-L1(CPS) (%) |  |  |  |  | 0.352 |  |  |  |  | 0.950 |
| ≥ 1 | 3 | 0.59 (0.50, 0.71) | 45.6 | <0.001 |  | 2 | 0.85 (0.70, 1.02) | 0.0 | 0.087 |  |
| < 1 | 3 | 0.67 (0.56, 0.81) | 43.5 | <0.001 |  | 2 | 0.86 (0.68, 1.08) | 0.0 | 0.191 |  |
| PD-L1(IC) (%) |  |  |  |  | 0.576 |  |  |  |  | 0.837 |
| ≥ 1 | 2 | 0.72 (0.45, 1.17) | 88.4 | <0.001 |  | 2 | 0.86 (0.73, 1.01) | 0.0 | 0.068 |  |
| < 1 | 1 | 0.84 (0.67, 1.05) | 0.0 | 0.120 |  | 1 | 0.89 (0.65, 1.21) | 0.0 | 0.460 |  |

Abbreviation: PFS, progression-free survival; OS, overall survival; ICIs, immune checkpoint inhibitors; HR, hazard ratio; CI, confidence interval; ECOG, eastern cooperative oncology group; RCC, renal cell carcinoma; ECOG, Eastern Cooperative Oncology Group performance status; CPS, combined positive score; IC, Immune Cell score
