# Supplementary material for: First-line immune checkpoint inhibitor plus anti-angiogenic therapy for advanced renal cell carcinoma: a meta-analysis of randomized controlled trials
Source: Front Pharmacol. 2026 Jul 27;17:1850724. doi: 10.3389/fphar.2026.1850724 (PMC13454646; doi:10.3389/fphar.2026.1850724)
Supplement: Supplementary file 9 [file Table4.docx]

**Supplementary Table 4**. Subgroup analysis of grade ≥3 TRAEs for ICIs combined with anti-angiogenic agents versus sunitinib in first-line treatment of advanced RCC, stratified by types of ICIs and anti-angiogenic agents.

| **Safety Outcome** | **Subgroup** | **Category** | **No. of Trials** | **OR (95% CI)** | **I^2^ (%)** | ***P* value** | **P for interaction** |
| --- | --- | --- | --- | --- | --- | --- | --- |
| Diarrhea | ICIs class | PD-1 | 3 | 1.90 (1.35, 2.69) | 0.0 | < 0.001 | 0.535 |
|  |  | PD-L1 | 3 | 1.16 (0.25, 5.34) | 87.5 | 0.851 |  |
|  | VEGF inhibitor class | multi-TKI | 3 | 1.72 (1.10, 2.67) | 0.0 | 0.017 | < 0.001 |
|  |  | anti-VEGF mAb | 1 | 0.35 (0.15, 0.85) | 0.0 | 0.020 |  |
|  |  | VEGFR-TKI | 2 | 2.49 (1.67, 3.70) | 0.0 | < 0.001 |  |
| Decreased appetite | ICIs class | PD-1 | 3 | 2.70 (1.24, 5.85) | 0.0 | 0.012 | 0.214 |
|  |  | PD-L1 | 3 | 0.98 (0.24, 3.96) | 65.1 | 0.979 |  |
|  | VEGF inhibitor class | multi-TKI | 3 | 2.32 (1.06, 5.10) | 0.0 | 0.036 | 0.019 |
|  |  | anti-VEGF mAb | 1 | 0.22 (0.05, 1.01) | 0.0 | 0.051 |  |
|  |  | VEGFR-TKI | 2 | 2.36 (0.90, 6.20) | 9.6 | 0.080 |  |
| Weight loss | ICIs class | PD-1 | 1 | 44.17 (2.66, 732.10) | 0.0 | 0.008 | 0.178 |
|  |  | PD-L1 | 2 | 4.84 (1.01, 23.24) | 0.0 | 0.049 |  |
|  | VEGF inhibitor class | multi-TKI | 2 | 8.62 (0.42, 177.43) | 62.7 | 0.163 | 0.969 |
|  |  | anti-VEGF mAb |  |  |  |  |  |
|  |  | VEGFR-TKI | 1 | 9.28 (1.17, 73.53) | 0.0 | 0.035 |  |
| Vomiting | ICIs class | PD-1 | 3 | 1.14 (0.37, 3.51) | 20.7 | 0.814 | 0.387 |
|  |  | PD-L1 | 3 | 0.43 (0.06, 2.93) | 46.6 | 0.388 |  |
|  | VEGF inhibitor class | multi-TKI | 3 | 2.00 (0.71, 5.62) | 0.0 | 0.189 | 0.018 |
|  |  | anti-VEGF mAb | 1 | 0.11 (0.01, 2.03) | 0.0 | 0.137 |  |
|  |  | VEGFR-TKI | 2 | 0.25 (0.07, 0.88) | 0.0 | 0.031 |  |
| Palmar-plantar erythrodysesthesia syndrome | ICIs class | PD-1 | 3 | 1.08 (0.75, 1.57) | 0.0 | 0.681 | 0.600 |
|  |  | PD-L1 | 3 | 0.70 (0.14, 3.42) | 84.0 | 0.659 |  |
|  | VEGF inhibitor class | multi-TKI | 3 | 1.24 (0.79, 1.96) | 13.5 | 0.351 | 0.004 |
|  |  | anti-VEGF mAb | 1 | 0.01 (0.00, 0.18) | 0.0 | 0.002 |  |
|  |  | VEGFR-TKI | 2 | 1.33 (0.86, 2.04) | 0.0 | 0.195 |  |
| ALT increased | ICIs class | PD-1 | 2 | 5.38 (3.05, 9.47) | 0.0 | < 0.001 | 0.095 |
|  |  | PD-L1 | 2 | 2.61 (1.39, 4.92) | 0.0 | 0.003 |  |
|  | VEGF inhibitor class | multi-TKI | 2 | 4.93 (2.16, 11.24) | 0.0 | <0.001 | 0.575 |
|  |  | anti-VEGF mAb |  |  |  |  |  |
|  |  | VEGFR-TKI | 2 | 3.48 (1.43, 8.51) | 69.5 | 0.006 |  |
| AST increased | ICIs class | PD-1 | 2 | 4.37 (2.18, 8.79) | 0.0 | < 0.001 | 0.064 |
|  |  | PD-L1 | 2 | 1.73 (0.86, 3.46) | 0.0 | 0.123 |  |
|  | VEGF inhibitor class | multi-TKI | 2 | 2.58 (0.96, 6.97) | 27.5 | 0.061 | 0.896 |
|  |  | anti-VEGF mAb |  |  |  |  |  |
|  |  | VEGFR-TKI | 2 | 2.82 (1.19, 6.67) | 50.5 | 0.018 |  |
| Hypertension | ICIs class | PD-1 | 3 | 1.25 (1.01, 1.56) | 1.2 | 0.042 | 0.679 |
|  |  | PD-L1 | 3 | 1.42 (0.82, 2.48) | 86.1 | 0.213 |  |
|  | VEGF inhibitor class | multi-TKI | 3 | 1.51 (1.08, 2.13) | 52.8 | 0.017 | 0.028 |
|  |  | anti-VEGF mAb | 1 | 0.80 (0.56, 1.16) | 0.0 | 0.238 |  |
|  |  | VEGFR-TKI | 2 | 1.44 (0.94, 2.22) | 72.5 | 0.098 |  |
| Proteinuria | ICIs class | PD-1 | 3 | 1.71 (0.97, 3.01) | 34.3 | 0.064 | 0.519 |
|  |  | PD-L1 | 2 | 2.95 (0.62, 14.14) | 74.7 | 0.175 |  |
|  | VEGF inhibitor class | multi-TKI | 3 | 1.91 (1.24, 2.93) | 0.0 | 0.003 | 0.055 |
|  |  | anti-VEGF mAb | 1 | 7.64 (1.74, 33.60) | 0.0 | 0.007 |  |
|  |  | VEGFR-TKI | 1 | 0.99 (0.44, 2.23) | 0.0 | 0.981 |  |
| Anemia | ICIs class | PD-1 | 3 | 0.16 (0.07, 0.38) | 0.0 | < 0.001 | 0.273 |
|  |  | PD-L1 | 3 | 0.08 (0.03, 0.19) | 0.0 | < 0.001 |  |
|  | VEGF inhibitor class | multi-TKI | 3 | 0.16 (0.08, 0.35) | 0.0 | < 0.001 | 0.273 |
|  |  | anti-VEGF mAb | 1 | 0.05 (0.01, 0.37) | 0.0 | 0.004 |  |
|  |  | VEGFR-TKI | 2 | 0.06 (0.02, 0.20) | 0.0 | < 0.001 |  |
| Thrombocytopenia | ICIs class | PD-1 | 3 | 0.03 (0.01, 0.14) | 0.0 | < 0.001 | 0.382 |
|  |  | PD-L1 | 2 | 0.07 (0.03, 0.21) | 8.6 | < 0.001 |  |
|  | VEGF inhibitor class | multi-TKI | 2 | 0.06 (0.01, 0.24) | 0.0 | < 0.001 | 0.301 |
|  |  | anti-VEGF mAb | 1 | 0.12 (0.04, 0.39) | 0.0 | 0.001 |  |
|  |  | VEGFR-TKI | 2 | 0.02 (0.00, 0.14) | 0.0 | < 0.001 |  |
| Neutropenia | ICIs class | PD-1 | 3 | 0.09 (0.04, 0.21) | 0.0 | < 0.001 | 0.455 |
|  |  | PD-L1 | 2 | 0.05 (0.02, 0.17) | 38.2 | < 0.001 |  |
|  | VEGF inhibitor class | multi-TKI | 2 | 0.11 (0.04, 0.32) | 21.9 | < 0.001 | 0.378 |
|  |  | anti-VEGF mAb | 1 | 0.11 (0.03, 0.49) | 0.0 | 0.004 |  |
|  |  | VEGFR-TKI | 2 | 0.04 (0.01, 0.13) | 0.0 | < 0.001 |  |
| Rash | ICIs class | PD-1 | 2 | 7.08 (1.87, 26.85) | 0.0 | 0.004 | 0.120 |
|  |  | PD-L1 | 3 | 1.78 (0.59, 5.42) | 0.0 | 0.308 |  |
|  | VEGF inhibitor class | multi-TKI | 3 | 4.90 (1.47, 16.32) | 0.0 | 0.010 | 0.533 |
|  |  | anti-VEGF mAb | 1 | 1.49 (0.25, 8.94) | 0.0 | 0.665 |  |
|  |  | VEGFR-TKI | 1 | 2.55 (0.49, 13.20) | 0.0 | 0.265 |  |
| Fatigue | ICIs class | PD-1 | 3 | 0.59 (0.37, 0.93) | 0.0 | 0.022 | 0.952 |
|  |  | PD-L1 | 3 | 0.57 (0.33, 0.99) | 75.2 | 0.047 |  |
|  | VEGF inhibitor class | multi-TKI | 3 | 0.66 (0.36, 1.19) | 0.0 | 0.165 | 0.247 |
|  |  | anti-VEGF mAb | 1 | 0.29 (0.11, 0.72) | 0.0 | 0.008 |  |
|  |  | VEGFR-TKI | 2 | 0.68 (0.41, 1.13) | 33.6 | 0.135 |  |

Abbreviation: TRAEs,treatment-related adverse events; TKI, tyrosine kinase inhibitor; mAb, monoclonal antibody; OR, odd ratio; CI, confidence interval; ICI, immune checkpoint inhibitor; RCC, renal cell carcinoma; VEGF, vascular endothelial growth factor; TKI, tyrosine kinase inhibitor; VEGFR, vascular endothelial growth factor receptor
