# Supplementary material for: First-line immune checkpoint inhibitor plus anti-angiogenic therapy for advanced renal cell carcinoma: a meta-analysis of randomized controlled trials
Source: Front Pharmacol. 2026 Jul 27;17:1850724. doi: 10.3389/fphar.2026.1850724 (PMC13454646; doi:10.3389/fphar.2026.1850724)
Supplement: Supplementary file 10 [file Table5.docx]

**Supplementary Table 5.** Sensitivity analyses by exclusion of each included trial.

| Exclusion of | PFS | | | OS | | | ORR | | | DCR | | | Grade 3 or worse TRAEs | | | TRAEs leading to discontinuation | | |
| --- | --- | --- | --- | --- | --- | --- | --- | --- | --- | --- | --- | --- | --- | --- | --- | --- | --- | --- |
|  | HR (95% CI) | I^2^ (%) | *P* | HR (95% CI) | I^2^ (%) | *P* | OR (95% CI) | I^2^ (%) | *P* | OR (95% CI) | I^2^ (%) | *P* | OR (95% CI) | I^2^ (%) | *P* | OR (95% CI) | I^2^ (%) | *P* |
| CheckMate 9ER | 0.63 (0.54, 0.75) | 77.2 | <0.001 | 0.83 (0.77, 0.91) | 12.5 | <0.001 | 3.03 (1.85, 4.95) | 93.3 | <0.001 | 1.87 (1.38, 2.53) | 70.9 | <0.001 | 1.15 (0.87, 1.53) | 79.4 | 0.315 | 3.38 (2.74, 4.17) | 20.1 | <0.001 |
| CLEAR | 0.66 (0.58, 0.74) | 62.1 | <0.001 | 0.83 (0.77, 0.90) | 13.6 | <0.001 | 2.90 (1.81, 4.65) | 92.6 | <0.001 | 1.89 (1.38, 2.59) | 73.3 | <0.001 | 1.13 (0.88, 1.46) | 75.4 | 0.344 | 3.06 (2.47, 3.78) | 0.0 | <0.001 |
| ETER100 | 0.64 (0.55, 0.75) | 75.7 | <0.001 | 0.84 (0.77, 0.91) | 0.0 | <0.001 | 2.65 (1.80, 3.90) | 89.6 | <0.001 | 1.93 (1.39, 2.68) | 77.8 | <0.001 | 1.24 (0.92, 1.68) | 82.8 | 0.155 |  |  |  |
| IMmotion151 | 0.60 (0.53, 0.67) | 56.7 | <0.001 | 0.81 (0.74, 0.88) | 0.0 | <0.001 | 3.60 (2.66, 4.87) | 80.9 | <0.001 | 2.24 (1.67, 3.02) | 66.0 | <0.001 | 1.36 (1.19, 1.54) | 44.7 | <0.001 | 3.48 (2.79, 4.35) | 10.2 | <0.001 |
| JAVELIN Renal 101 | 0.62 (0.52, 0.73) | 78.4 | <0.001 | 0.81 (0.74, 0.89) | 4.8 | <0.001 | 3.06 (1.84, 5.08) | 93.3 | <0.001 | 2.11 (1.44, 3.07) | 80.2 | <0.001 | 1.21 (0.88, 1.26) | 82.9 | 0.232 | 3.36 (2.71, 4.16) | 21.1 | <0.001 |
| KEYNOTE-426 | 0.61 (0.52, 0.73) | 77.6 | <0.001 | 0.82 (0.75, 0.90) | 15.6 | <0.001 | 3.22 (1.94, 5.33) | 93.2 | <0.001 | 2.13 (1.47, 3.11) | 79.6 | <0.001 | 1.22 (0.89, 1.67) | 83.0 | 0.219 |  |  |  |
| RENOTORCH | 0.62 (0.53, 0.73) | 78.6 | <0.001 | 0.84 (0.77, 0.90) | 0.0 | <0.001 | 3.09 (1.91, 5.00) | 93.4 | <0.001 | 2.14 (1.51, 3.03) | 79.6 | <0.001 | 1.23 (0.91, 1.66) | 83.0 | 0.171 | 3.44 (2.83, 4.18) | 0.0 | <0.001 |

Abbreviation: PFS, progression free survival; OS, overall survival; ORR, objective response rate; DCR, disease control rate; TRAEs, treatment-related adverse events; HR, hazard ratio; CI, confidence interval; OR, odd ratios
