# Supplementary material for: First-line immune checkpoint inhibitor plus anti-angiogenic therapy for advanced renal cell carcinoma: a meta-analysis of randomized controlled trials
Source: Front Pharmacol. 2026 Jul 27;17:1850724. doi: 10.3389/fphar.2026.1850724 (PMC13454646; doi:10.3389/fphar.2026.1850724)
Supplement: Supplementary file 11 [file Table6.docx]

**Supplementary Table 6.** Post-Progression Treatment Landscape and Crossover Characteristics Across Seven Phase RCTs in First-Line Advanced RCC

| **Item** | **CheckMate 9ER** | **CLEAR** | **ETER100** | **IMmotion151** | **JAVELIN**  **Renal 101** | **KEYNOTE-426** | **RENOTORCH** |
| --- | --- | --- | --- | --- | --- | --- | --- |
| Trial Identification |  |  |  |  |  |  |  |
| Data cutoff (year) | 2024 | 2022 | 2024 | 2020 | 2023 | 2023 | 2023 |
| Median follow-up (months) | 67.6 | 49.8 | 22.8 | > 40.0 | 73.7 | 67.2 | 14.6 |
| OS data maturity | Final analysis | Final (prespecified) | Interim (immature) | Final analysis | Final analysis | Final analysis | Interim (immature) |
| Post-Progression Treatment: Sunitinib arm |  |  |  |  |  |  |  |
| Any subsequent therapy, n | 168/305 (55.1%) | 246/357 (68.9%) | 135/263 (51.3%) | NR | 308/444 (69.4%) | 300/406 (73.9%) | 71/140 (50.7%) |
| Subsequent ICI-based therapy, n | 136/168 (81.0%) | 195/357 (54.6%) | 61/263 (23.2%) | NR | 238/444 (53.6%) | 240/300 (80.0%) | 37/140 (26.4%) |
| Subsequent TKI therapy, n | 86/168 (51.2%) | 162/357 (45.4%) | 113/263 (43.0%) | NR | 205/444 (46.2%) | 216/300 (72.0%) | 60/140 (42.9%) |
| Most common subsequent regimens | Nivolumab (75.0%), Cabozantinib (29.8%), Axitinib (16.7%) | Nivolumab (49.0%), Cabozantinib (30.0%), Axitinib (13.2%) | TKI (43.0%), ICI (23.2%) | NR | Nivolumab (50.5%), Cabozantinib (26.6%), Everolimus (13.3%) | PD-(L)1 inhibitor (80.0%), VEGFR inhibitor (72.0%), mTOR inhibitor (16.7%) | VEGFR-TKI (42.9%), ICI/anti-PD-1 (26.4%) |
| Post-Progression Treatment: Combination arm |  |  |  |  |  |  |  |
| Any subsequent therapy, n | 129/299 (43.1%) | 181/355 (51.0%) | 68/264 (25.8%) | NR | 257/442 (58.1%) | 237/381 (62.2%) | 34/93 (36.6%) |
| Subsequent ICI-based therapy, n | 46/129 (35.7%) | 56/355 (15.8%) | 25/264(9.5%) | NR | 83/442 (18.8%) | 63/237 (26.6%) | 11/93 (11.8%) |
| Subsequent TKI therapy, n | 102/129 (79.1%) | 163/355 (45.9%) | 52/264 (20.0%) | NR | 226/442 (51.1%) | 206/237 (86.9%) | 31/93 (33.3%) |
| Most common subsequent regimens | Axitinib (27.9%), Sunitinib (19.4%), Lenvatinib (18.6%) | Cabozantinib (28.5%), Axitinib (12.1%), Nivolumab (11.8%) | TKI (20.0%), ICI (9.5%) | NR | Cabozantinib (33.9%), Nivolumab (15.2%), Everolimus (14.3%) | VEGFR inhibitor (86.9%), mTOR inhibitor (29.1%), PD-(L)1 inhibitor (26.6%) | VEGFR-TKI (33.3%), ICI/anti-PD-1 (11.8%) |
| Crossover and Protocol Design |  |  |  |  |  |  |  |
| Formal crossover permitted | No | No | NR | No | No | No | No |
| Crossover rate (SUN -combination), n | N/A (no formal crossover) | NR | NR | NR | NR | NR | NR |
| ICI use in control arm post-progression | Yes (81.0%) | Yes (54.6%) | Yes (23.2%) | NR | Yes (53.6%) | Yes (80.0%) | Yes (26.4%) |

Abbreviations: ICI, immune checkpoint inhibitor; NR, not reported; TKI, tyrosine kinase inhibitor
